# Global, regional, and national burdens of atrial fibrillation/flutter from 1990 to 2019: An age-period-cohort analysis using the Global Burden of Disease 2019 study

**DOI:** 10.7189/jogh.13.04154

**Published:** 2023-11-22

**Authors:** Xiaofei Li, Zeye Liu, Xianchao Jiang, Ruibing Xia, Yakun Li, Xiangbin Pan, Yan Yao, Xiaohan Fan

**Affiliations:** 1 Arrhythmia Center, Fuwai Hospital, National Center for Cardiovascular Diseases, Chinese Academy of Medical Sciences, Peking Union Medical College, Beijing, China; 2 Department of Cardiac Surgery, Fuwai Hospital, Chinese Academy of Medical Sciences and Peking Union Medical College, Beijing, China; 3 Department of Medicine I, University Hospital Munich, Ludwig-Maximilians-University Munich (LMU), Munich, Germany; 4 Laboratory of Experimental Intensive Care and Anesthesiology, Academic Medical Center, Amsterdam, the Netherlands

## Abstract

**Background:**

Atrial fibrillation/flutter (AF/AFL) significantly impacts countries with varying income levels. We aimed to present worldwide estimates of its burden from 1990 to 2019 using data from the Global Burden of Disease (GBD) study.

**Methods:**

We derived cause-specific AF/AFL mortality and disability-adjusted life-year (DALY) estimates from the GBD 2019 study data. We used an age-period-cohort (APC) model to predict annual changes in mortality (net drifts), annual percentage changes from 50-55 to 90-95 years (local drifts), and period and cohort relative risks (period and cohort effects) between 1990 and 2019 by sex and sociodemographic index (SDI) quintiles. This allowed us to determine the impacts of age, period, and cohort on mortality and DALY trends and the inequities and treatment gaps in AF/AFL management.

**Results:**

Based on GBD data, our estimates showed that 59.7 million cases of AF/AFL occurred worldwide in 2019, while the number of AF/AFL deaths rose from 117 000 to 315 000 (61.5% women). All-age mortality and DALYs increased considerably from 1990 to 2019, and there was an increase in age risk and a shift in death and DALYs toward the older (>80) population. Although the global net drift mortality of AF/AFL decreased overall (-0.16%; 95% confidence interval (CI) = -0.20, 0.12 per year), we observed an opposite trend in the low-middle SDI (0.53%; 95% CI = 0.44, 0.63) and low SDI regions (0.32%; 95% CI = 0.18, 0.45). Compared with net drift among men (-0.08%; 95% CI = -0.14, -0.02), women had a greater downward trend or smaller upward trend of AF/AFL (-0.21%; 95% CI = -0.26, -0.16) in mortality in middle- and low-middle-SDI countries (*P* < 0.001). Uzbekistan had the largest net drift of mortality (4.21%; 95% CI = 3.51, 4.9) and DALYs (2.16%; 95% CI = 2.05, 2.27) among all countries. High body mass index, high blood pressure, smoking, and alcohol consumption were more prevalent in developed countries; nevertheless, lead exposure was more prominent in developing countries and regions.

**Conclusions:**

The burden of AF/AFL in 2019 and its temporal evolution from 1990 to 2019 differed significantly across SDI quintiles, sexes, geographic locations, and countries, necessitating the prioritisation of health policies based on risk-differentiated, cost-effective AF/AFL management.

Due to global population aging, atrial fibrillation/flutter (AF/AFL) has become the most prevalent persistent type of arrhythmia worldwide and a leading cause of systemic embolism, particularly ischemic stroke, heart failure, and other cardiovascular illness [1,2], resulting in direct and indirect family and societal costs. The Global Burden of Diseases (GBD) study 2017 reported on the global burden of AF/AFL, but did not distinguish between the relative contributions of period, age, and cohort to the impact of mortality of AF/AFL. There is also a lack of reliable evidence on changing trends in the main variable risk factors for AF/AFL in different countries and regions, which is key to developing effective prevention and treatment strategies. There is a need to analyse mortality and disability-adjusted life-years (DALYs) in different areas and identify the changing trends of risk factors, which would provide relevant epidemiological data on AF/AFL trends and inform medical resource investment.

The mortality risk for patients with AF/AFL, which can be categorised by period, age, and birth cohort effects, depends on biological age (age effect), but also varies among birth cohorts due to economic and social developmental factors and new diagnostic and therapeutic choices. Thus, an analysis of mortality and DALY trends focused on the effects of these three variables could help with identifying the successes and shortcomings of current treatments and inform future improvements.

To the best of our knowledge, this is the first study to use updated GBD 2019 data and age-period-cohort models to explore changes in AF/AFL mortality across 204 countries and territories from 1990 to 2019. It was produced as part of the GBD Collaborator Network and in accordance with GBD Protocols (Contact ID: 0034o00001nHH4NAAW).

## METHODS

### GBD 2019 overview

The GBD 2019 provides the most up-to-date descriptive epidemiological data on a total of 369 diseases and injuries for 204 countries and territories from 1990 to 2019. Each death in the GBD is assigned to a single underlying cause from a mutually and collectively exhaustive list of diseases and injuries. The study team obtained ethics approval from the University of Washington Institutional Review Board Committee, while the protocol and all data are available online upon request from the EPHI-IHME Office [3] or from the GBD 2019 database, which contains the participants’ deanonymised data [4].

### Data sources

We mapped cause-specific deaths attributed to AF/AFL, diagnosed by electrocardiography as per previous GBD studies [5,6], to the GBD cause list per the International Classification of Diseases and Injuries (ICD) codes 472.3-427.32 (ICD-9) and 148-148.92 (ICD-10). Data inputs, processing, synthesis, and final models are available elsewhere [7]. The GBD network used standardised tools within a Bayesian framework to leverage all available data across time, age, geography, and across causes and domains of health to generate disease estimates, allowing for the “borrowing” of information from the available data to produce burden estimates for countries worldwide without primary data sources. All disease estimates from the GBD contain 95% uncertainty intervals (UIs) for every metric, which are based on the 25th and 975th ordered values of 1000 draws of the posterior distribution [7]. Countries with fewer or no data sources generally have larger 95% UIs, suggesting greater inaccuracy in disease estimates. The GBD study uses deidentified data and obtained a waiver of informed consent from the University of Washington Institutional Review Board.

We used the 2019 sociodemographic index (SDI) to categorise each country into one of five quintiles. SDI is an indicator estimated as a composite of income per capita, average years of schooling, and the fertility rate among women under 25 years of age [7]. It is scaled from 0 to 1, with higher values indicating a higher socioeconomic level.

### Statistical analysis

We aimed to examine the global trends of AF/AFL mortality and DALYs. To do so, we first assessed temporal trends by all-age mortality (crude mortality) and age-standardised mortality and the relative change in mortality as a percentage between 1990 and 2019. We calculated age-standardised mortality by using global age-standardised population data from the GBD 2019 [7] and examined the age distribution of deaths as an indirect indicator of survival by arranging death counts in six age strata (0-49, 50-59, 60-69, 70-79, 80-89, >90) and calculating the proportions of deaths from each age stratum.

We then used the age-period-cohort (APC) model to analyse the underlying trends in mortality by age, period, and birth cohort [8]. This model is designed to unpack the contributions of age-associated biological factors and technological and social factors on disease trends, extending beyond traditional epidemiological analyses. It has been adopted in descriptive epidemiological studies for certain chronic cardiovascular diseases [9]. Generally, the APC model fits a log-linear Poisson model over a Lexis diagram of observed rates and quantifies the additive effects of age, period, and birth cohorts. As the relationship among age, period, and cohort is perfectly linear (birth cohort = period − age), it is statistically impossible to estimate their independent effects, which is known as the identification problem [10]. We circumvented this issue by producing estimable APC parameters and functions without imposing arbitrary constraints on model parameters [11]. The APC model was designed using open-source tools in R, version 4.2.2., as detailed elsewhere [12].

We used GBD 2019 mortality estimates for AF/AFL and the population data of each country/region as data inputs for the APC model. In such cases, the age and period intervals usually must all be equal, i.e. five-year age groups should be used with five-year calendar periods. As GBD estimates are produced in an unequally spaced data format (five-year age groups with annual data), we arranged the data into a single unit framework by selecting the death and population counts from the mid-year of six five-year periods ((1992) 1990-1994, (1997) 1995-1999… (2017) 2015-2019) to represent the specific period. The input data included nine age groups (from 55-65 to 90-95 in five-year age group intervals) and partially overlapping ten-year birth cohorts, as referenced by the mid-year of birth, from 1895 to 1905 (the 1900 cohort) to 1955 to 1965 (the 1960 cohort). The fitted APC model estimated the overall temporal trend in mortality, which is expressed as the annual percentage change in mortality (i.e. the net drift of mortality, % per year). Technically, the net drift is determined by two components: the trend attributable to calendar time and another attributable to the successive cohorts. The APC model also estimated the temporal trend of mortality within each age group, expressed as the annual percentage age change in age-specific mortality (i.e. the local drift of mortality, % per year), reflecting trends in the birth cohort effect. A drift of ±1% per year or more is considered a substantial change in mortality [12] because this approximates ±10%, ±18%, and ±26% of the change in the fitted rate throughout 10, 20, and 30 years, respectively. We tested the significance of trends in annual percentage changes with a Wald χ^2^ test [12]. The APC model outputs also included fitted longitudinal age-specific rates in the referent cohort adjusted for period deviations to represent age-associated natural history (i.e. age effects) and period (cohort) relative risks of mortality for each period (cohort) to represent period (cohort) effects [12]. The relative risk is computed as the ratio of age-specific rates in each period (cohort) relative to the reference period (cohort). Both period (cohort) rate ratio curves incorporate the entire value of the net drift. The choice of referent period (cohort) is arbitrary and does not affect the interpretation of the results. Statistical tests were two-sided, and *P* < 0.05 was considered significant. We conducted all analyses in R, version 4.2.2. (R Core Team, Vienna, Austria) [8].

## RESULTS

### Global and regional trends in AF/AFL mortality and DALYs, 1990-2019

**Figure 1:**
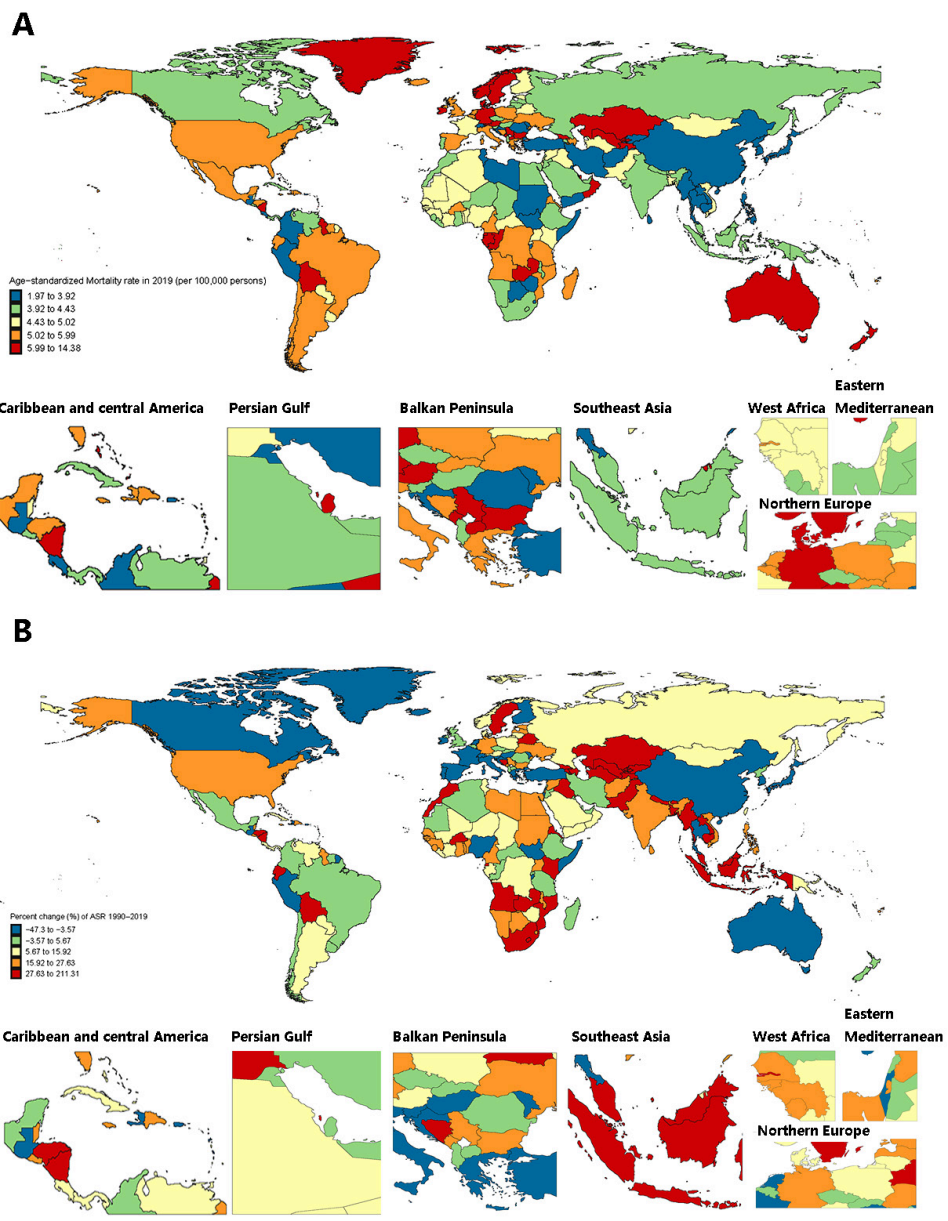
Age-standardised mortality. **Panel A.** Percentage change in mortality. **Panel B.** Rates of atrial fibrillation/atrial flutter across 204 countries and territories from 1990 to 2019. In this figure, the ASR and percentage change in ASR in North America all increased. This result may be due to the high overall life expectancy of the population in the region and the high burden of disease as a result of the region's aging population. ASR – age-standard mortality rate.

In the past 30 years, the global population grew by 45% from 5.3 billion (95% UI = 5.2, 5.5) to 7.7 billion (95% UI = 7.5, 8.0), while the number of AF/AFL deaths has increased by 169% from 117 000 (103 000-138 000) to 315 000 (61.5% women) (95% UI = 267 900, 355 000). In 2019, the global death rate for AF/AFL was 4.08 (3.46, 4.67) per 100 000 people, representing an increase of 86.0 from 1990 (69.0, 100.0), although the age-standardised mortality rate remained comparable. Meanwhile, all-age mortality rates were generally lower than age-standardised mortality rates in all except for high SDI regions, indicating they more appropriately capture the true burden of AF/AFL for high-income countries. The percentage change in all-age mortality had an inverted U-shape across different SDIs, with the greatest increase in middle-SDI (141%; 95% UI = 107, 177) and low-middle-SDI regions (133%; 95% UI = 89, 180) and the smallest in low-SDI (36%; 95% UI = 13, 70) and high-SDI regions (89%; 95% UI = 69, 101). However, the APC model estimated a moderate decreasing net drift of AF/AFL mortality of -0.16% (95% CI = -0.20, -0.12) per year globally, attributable to high-middle-SDI (-0.56%; 95% CI = -0.63, -0.49) and high SDI regions (-0.19%; 95% CI = -0.27, -0.11). We found an upward trend in the net drift of AF/AFL mortality in middle- (0.05%; 95% CI = -0.02%, 0.12%) to low-middle-SDI regions (0.053%; 95% UI = 0.44, 0.63), likewise in the form of an inverted U-shape. Similarly, countries and areas with a high- or high-middle-SDI had higher all-age DALYs and age-standardised DALYs in 2019, with similar inverted U-shaped trends for the percentage change in DALYs and all-age DALYs. However, net drifts of DALYs decreased or changed slightly in high- and high-middle-SDI nations and regions, but they increased in middle- and low-SDI countries. The disparity between the net drift and the all-age/age-standardised drift may be attributable to variations in population composition and the multiple repercussions of the level of economic development (**Table 1**, **Figure 1**¸and Table S1 in the **Online Supplementary Document**).

**Table 1:** Trends in AF/AFL mortality across socio-demographic index quintiles, 1990-2019*

	Global		High SDI			High-middle SDI		Middle SDI		Low-middle SDI		Low SDI	
	**1990**	**2019**	**1990**	**2019**		**1990**	**2019**	**1990**	**2019**	**1990**	**2019**	**1990**	**2019**
**Deaths**													
n ×1000 (95% UI)*	117.04 (103.7, 138.45)	315.34 (267.96, 361.01)	47.16 (40.2, 59.6)	109.93 (86.25, 132.03)		33.99 (30.15, 43.29)	83.84 (70.92, 100.86)	20.78 (18.35, 23.54)	69.79 (59.85, 80.80)	10.54 (8.36, 12.76)	38.42 (32.14, 44.62)	4.5 (3.02, 5.73)	13.16 (9.59, 16.01)
Percentage of global (95% CI)	100.00	100.00	40.29		34.86	29.04	26.59	17.75	22.13	9.01	12.18	3.85	4.17
Percentage change of deaths 1990-2019 (95% CI)	169 (144, 189)		133 (108, 148)			147 (123, 166)		236 (189, 286)		264 (195, 337)		192 (143, 262)	
**All-age mortality rate**													
Rate per 100 000 (95% CI)	2.19 (1.94, 2.59)	4.08 (3.46, 4.67)	5.74 (4.89, 7.25)		10.85 (8.51, 13.03)	2.95 (2.62, 3.76)	5.86 (4.96, 7.05)	1.21 (1.07, 1.37)	2.91 (2.5, 3.37)	0.93 (0.74, 1.13)	2.18 (1.82, 2.53)	0.85 (0.57, 1.09)	1.17 (0.85, 1.42)
Percentage change of rate 1990-2019 (95% CI)	86 (69, 100)		89 (69, 101)			98 (79, 114)		141 (107, 177)		133 (89, 180)		36 (13, 70)	
**Age-standardised mortality rate**													
Rate per 100 000 (95% CI)	4.29 (3.73, 5.09)	4.38 (3.7, 5.05)	4.62 (3.93, 5.82)		4.61 (3.67, 5.52)	4.64 (4.04, 5.92)	4.47 (3.78, 5.39)	3.82 (3.32, 4.28)	4.11 (3.5, 4.75)	3.46 (2.66, 4.14)	4.21 (3.5, 4.87)	3.73 (2.42, 4.76)	4.3 (3.08, 5.25)
Percentage change of rate 1990-2019 (95% CI)	2 (-6, 9)		-0.2 (-11, 6)			-4 (-12, 3)		8 (-7, 25)		22 (-1, 46)		16 (-5, 42)	
**APC model estimates – net drift of mortality, % per year (95% UI)†**	-0.16 (-0.2, -0.12)		-0.19 (-0.27, -0.11)			-0.56 (-0.63, -0.49)		0.05 (-0.02, 0.12)		0.53 (0.44, 0.63)		0.32 (0.18, 0.45)	

AF/AFL – atrial fibrillation/ atrial flutter, SDI – socio-demographic index, APC – age-period-cohort

*All-age mortality = crude mortality rate. The age-standardised mortality rate is computed by direct standardisation with the global standard population in GBD 2019. Parentheses for all GBD health estimate indicate 95% uncertainty intervals; parentheses for net drift indicate 95% confidence intervals.

†Net drifts are estimates derived from the age-period-cohort model and denotes overall annual percentage change in mortality, which captures the contribution of the effects from calendar time and successive birth cohorts.

### National trends in AF/AFL mortality and DALYs, 1990-2019

AF/AFL mortality and DALY changes over the past 30 years varied greatly among nations and regions (**Figure 1**, Panel B, Figure S1, Panel B, and Tables S2-S3 in the **Online Supplementary Document**). A higher SDI did not appear to be related to reduced AF/AFL deaths or DALYs. Among 204 countries and territories, 130 had at least 100 deaths in 2019 attributed to AF/AFL, with China (n = 51 700; 95% UI = 43 600, 60 100), the USA (n = 33 200; 95% UI = 26 700, 40 300), India (n = 29 200; 95% UI = 22 600, 37 300), Germany (n = 18 200; 95% UI = 14 800, 22 500) and Japan (n = 13 100; 95% UI = 9800, 18 300) leading and accounting for 46.1% of AF/AFL deaths worldwide. Seventy-nine of these 130 countries demonstrated an upward trend (net drift 0.0% per year) in mortality, especially low, low-middle, and middle-SDI countries (75.9%, n/N = 60/79). Uzbekistan had the highest increase in AF/AFL-related all-age mortality, with a net drift of mortality of 4.21% (95% UI = 3.51, 4.90) per year. In 2019, 48 countries had a 2-fold all-age mortality rate compared to the global average, and four (Montenegro, Qatar, Bahrain, Saint Kitts and Nevis, and Greenland) had an age-standardised mortality rate of more than twice the global average; these countries were all high-middle or high SDI nations. Highly populous countries with notable increases in the number of AF/AFL deaths and percentage change of AF/AFL deaths were:

− Low SDI countries: Bangladesh (n = 4163) (343.78%; 95% UI = 211.59, 508.4) and Pakistan (n = 2712) (99.62%; 95% UI = 46.62, 176.56);
− Low-middle SDI countries: India (n = 29 155) (360.07%; 95% UI = 238.00, 500.24) and Vietnam (n = 3124) (177.95%; 95% UI = 92.52, 283.93);
− Middle SDI countries: China (n = 51 748) (218.43%; 95% UI = 155.11, 295.75) and Indonesia (n = 4992) (230.16%; 95% UI = 163.00, 303.54);
− High-middle SDI countries: Lebanon (n = 196) (237.24%; 95% UI = 118.03, 383.57) and Italy (n = 10 569) (115.19%; 95% UI = 79.63, 136.37);
− High SDI countries/regions: Singapore (n = 135) (253.91%; 95% UI = 78.10, 310.52), Republic of Korea (n = 2022) (333.56%; 95% UI = 55.12, 422.72), and Taiwan (Province of China) (n = 1399) (442.06%; 95% UI = 335.53, 594.13).

Similar trends were reported for DALYs. India, the USA, Germany and the Russian Federation accounted for 51.9% of all AF/AFL DALYs worldwide, while Uzbekistan showed the largest increase in AF/AFL-related all-age DALYs, with a net drift of DALYs of 2.16% (95% UI = 2.05, 2.27) per year (Table S3 in the **Online Supplementary Document**).

### Time trends in AF/AFL mortality across different age groups

**Figure 2:**
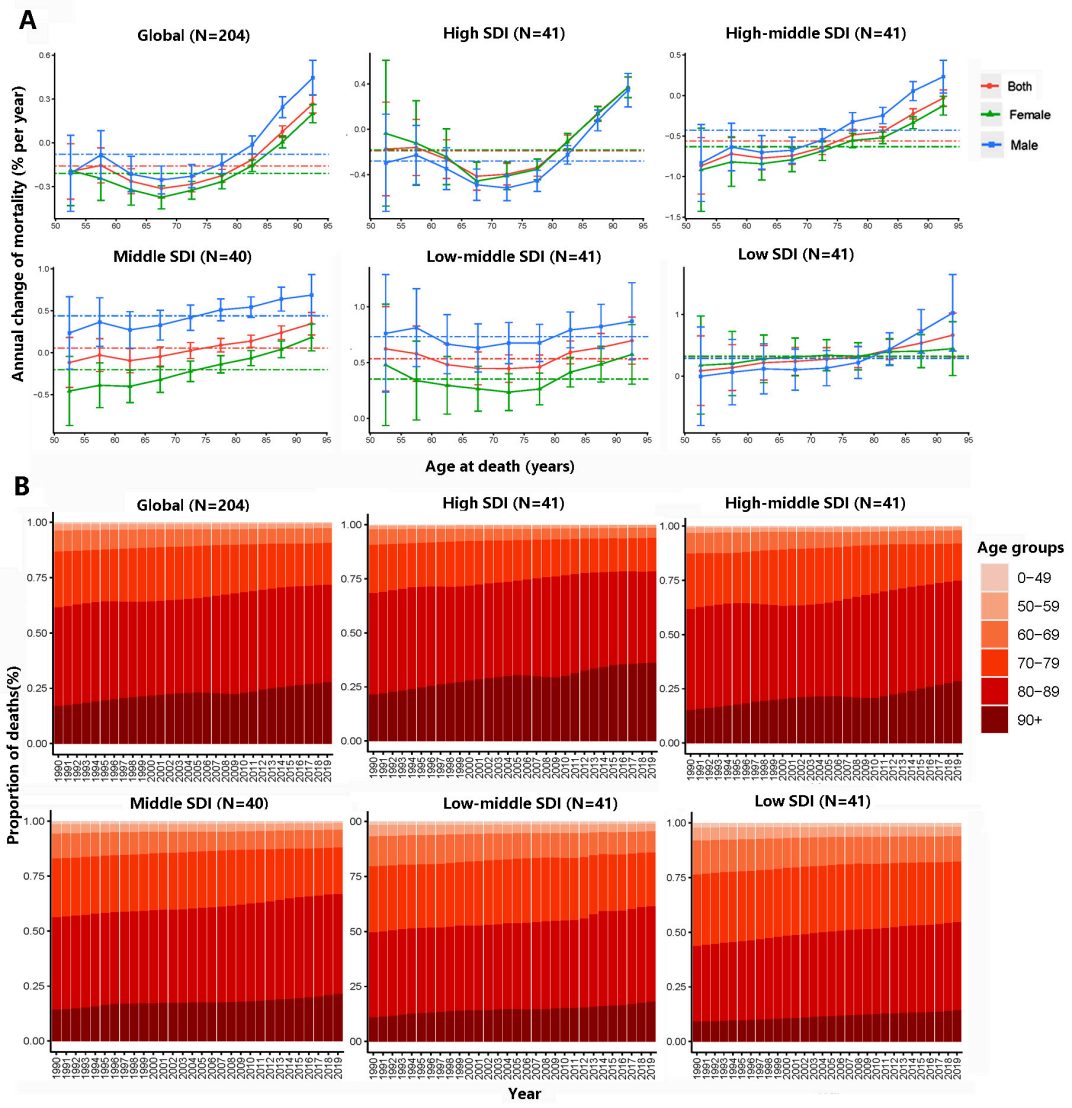
Local drifts of AF/AFL mortality and age distribution of death from AF/AFL by SDI quintiles, 1990-2019. **Panel A.** Local drifts of AF/AFL mortality (estimates from age-period-cohort models) for nine age groups (50-54 to 90-95 years), 1990-2019. **Panel B.** Temporal change in the relative proportion of AF/AFL deaths across age groups (0-49, 50-59, 60-69, 70-79, 80-89, >90 years), 1990-2019. AF/AFL – atrial fibrillation/atrial flutter. SDI – sociodemographic index.

The trends of AF/AFL mortality and DALYs varied greatly among different age groups, especially when comparing countries/regions with high and low SDIs (**Figure 2**). Globally, AF/AFL mortality was reduced among those aged <85 years and increased among elderly individuals; comparable trends were observed in countries with high- and high-middle-SDIs. The steepest mortality increase occurred in the 91-95 age group (0.27% per year; 95% CI = 0.20, 0.33). In middle-SDI countries, the turning point was approximately 70 years of age. However, in low-middle- and low-SDI countries, mortality attributable to AF/AFL increased across all age groups, particularly among elderly individuals. Men had much slower mortality declines than women, except for nations with a high SDI. Globally, most AF/AFL deaths occurred among those aged ≥80 years. There was a transition trend of death from the younger population (<80) to the older population (>80), particularly among those aged >90, and this trend was more pronounced in high and high-middle-SDI countries (**Figure 2**, Panel B). In countries with a high- or high-middle-SDI, AF/AFL accounted for a small number of deaths among people <60 years of age; in those with a low SDI, this proportion reached up to 7.3%. We observed similar trends of local drifts and temporal changes in DALYs among different age groups (Figure S2 in the **Online Supplementary Document**). Regarding sexual differences, compared with men (-0.08%; 95% CI = -0.14, -0.02), women had a greater downward trend or smaller upward trend of AF/AFL (net drift) deaths in middle- and low-middle-SDI countries/regions (-0.21; 95% CI = -0.26, -0.16) (*P* < 0.001). In the DALY analysis, we only found this trend in middle-SDI countries/regions (*P* < 0.001).

### Age, period, and cohort effects on AF/AFL mortality and DALYs

**Figure 3:**
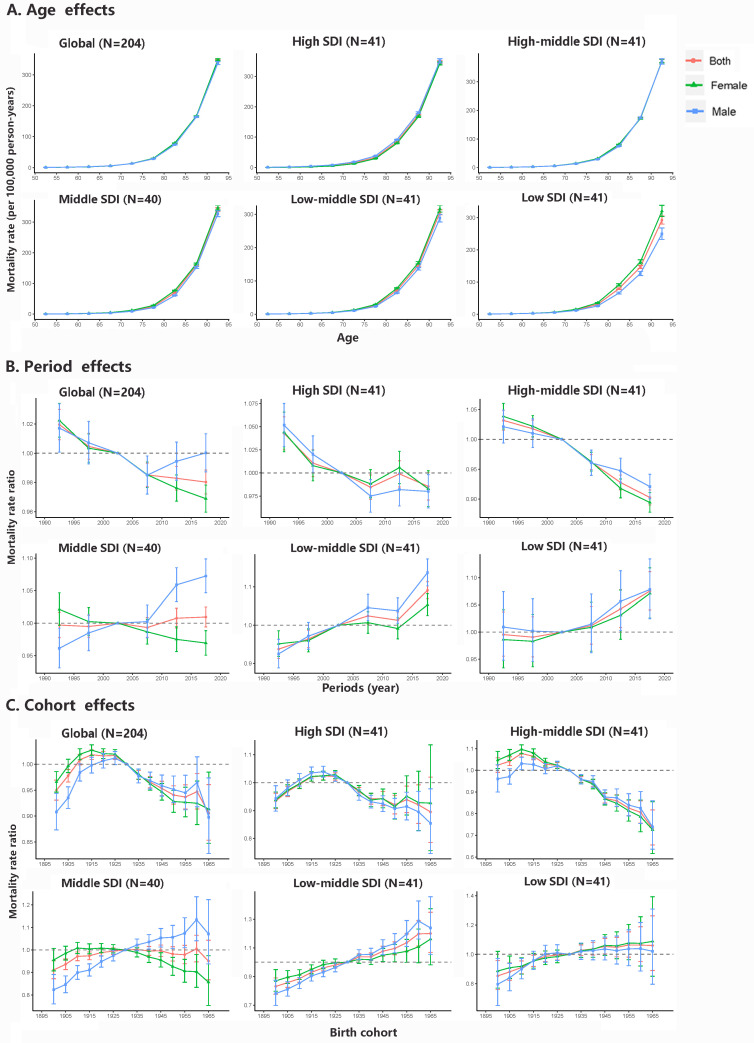
Age, period and cohort effects on AF/AFL mortality by SDI quintiles. **Panel A.** Age effects are shown by the fitted longitudinal age curves of mortality (per 100 000 person-years) adjusted for period deviations. **Panel B.** Period effects are shown by the relative risk of mortality (mortality rate ratio) and computed as the ratio of age-specific rates from 1990 to 1994 to 2015-2019 (the referent period, 2000-2004). **Panel C.** Cohort effects are shown by the relative risk of mortality and computed as the ratio of age-specific rates from the 1895 cohort to the 1965 cohort, with the reference cohort set at 1930. We presented the mortality rates or rate ratios and their corresponding 95% CIs. AF/AFL – atrial fibrillation/atrial flutter, SDI – sociodemographic index.

The APC model depicts the differences in mortality and DALY in different dimensions across different SDI countries or regions, particularly for period and cohort effects. In general, we observed comparable patterns of age effects were observed across all SDI quintiles, with the highest risk occurring in the group aged ≥90 years, and the risk rising with age, but no discernible differences in mortality by sex between SDI quintiles (**Figure 3**). Nonetheless, the period effect varied considerably among SDI quintiles. The risk of AF/AFL mortality decreased in nations with a high or high-middle-SDI, but increased in those with a low or low-middle-SDI. For middle-SDI countries, the overall period effects have remained practically constant over the past three decades, although the female population has shown a decline in mortality risk. The birth cohort effect revealed comparable downward trends in high- and high-middle-SDI countries and upward trends in low and low-middle-SDI countries. Compared to mortality, we found similar age and cohort effects across SDI quintiles; specifically for the period cohort, we observed an upward trend in DALYs across different SDI quintiles, except for high-middle-SDI countries (Figure S3 in the **Online Supplementary Document**).

### Age, period, and cohort effects in exemplary countries

**Figure 4:**
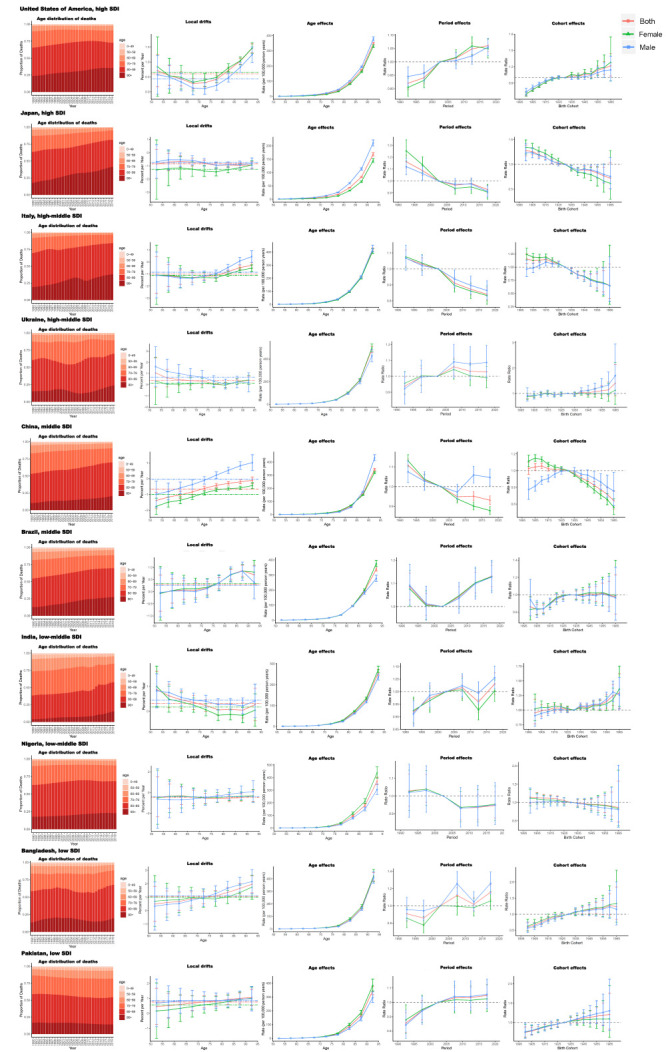
Age distribution of AF/AFL death and age-period-cohort effects on exemplar countries across SDI quintiles. AF/AFL – atrial fibrillation/atrial flutter, SDI – sociodemographic index.

We presented several exemplary countries across SDI quintiles to better characterise the major trends in AF/AFL mortality and DALYs by age-period-cohort effects worldwide (**Figure 4** and Figure S4 in the **Online Supplementary Document**).

As typical representative high-SDI countries, the USA and Japan exhibited entirely distinct patterns, with a general shift in AF/AFL mortality toward the older (>80) age group and an increase in risk by age, but different local drift, period effect, and birth cohort effects. As a well-developed East Asian country, Japan exhibited a downward trend across all age groups, with local drifts of less than 0% and a district period effect and cohort effect of decreasing scale. Conversely, for the USA, a typical industrialised North American nation, we observed a U-shaped increase across all age groups, particularly in the elderly and young populations, and upward trends for period and cohort effects. Italy, a country with a high-middle-SDI, had APC effects comparable to those of Japan, while Ukraine, a country with a high SDI, was similar to the USA, excluding the age distribution, as fewer individuals aged ≥90 years died from AF/AFL. China and Brazil are representative middle-SDI countries with a similar APC effect and a shift in AF/AFL deaths to the elderly population. China, the most populous middle-SDI nation, exhibited a downward mortality trend with a local drift of less than 0%, which was primarily attributable to the female population. Deaths attributable to AF/AFL in Brazil, however, exhibited an upward trend, with the local drift generally exceeding 0% and increasing with age. Among low-middle and low-SDI countries, Nigeria had a greater number of AF/AFL deaths in the elderly population (>90) and a downward trend of mortality, in contrast to India, Bangladesh, and Pakistan, which all had an upward trend of AF/AFL deaths across all age groups, as well as greater period and birth cohort effects. We saw similar APC effects between mortality and DALYs across different SDI countries (Figure S4 in the **Online Supplementary Document**).

### Percentage contributions of major risk factors to age-standardised death and DALYs of AF/AFL, 1990-2019

**Figure 5:**
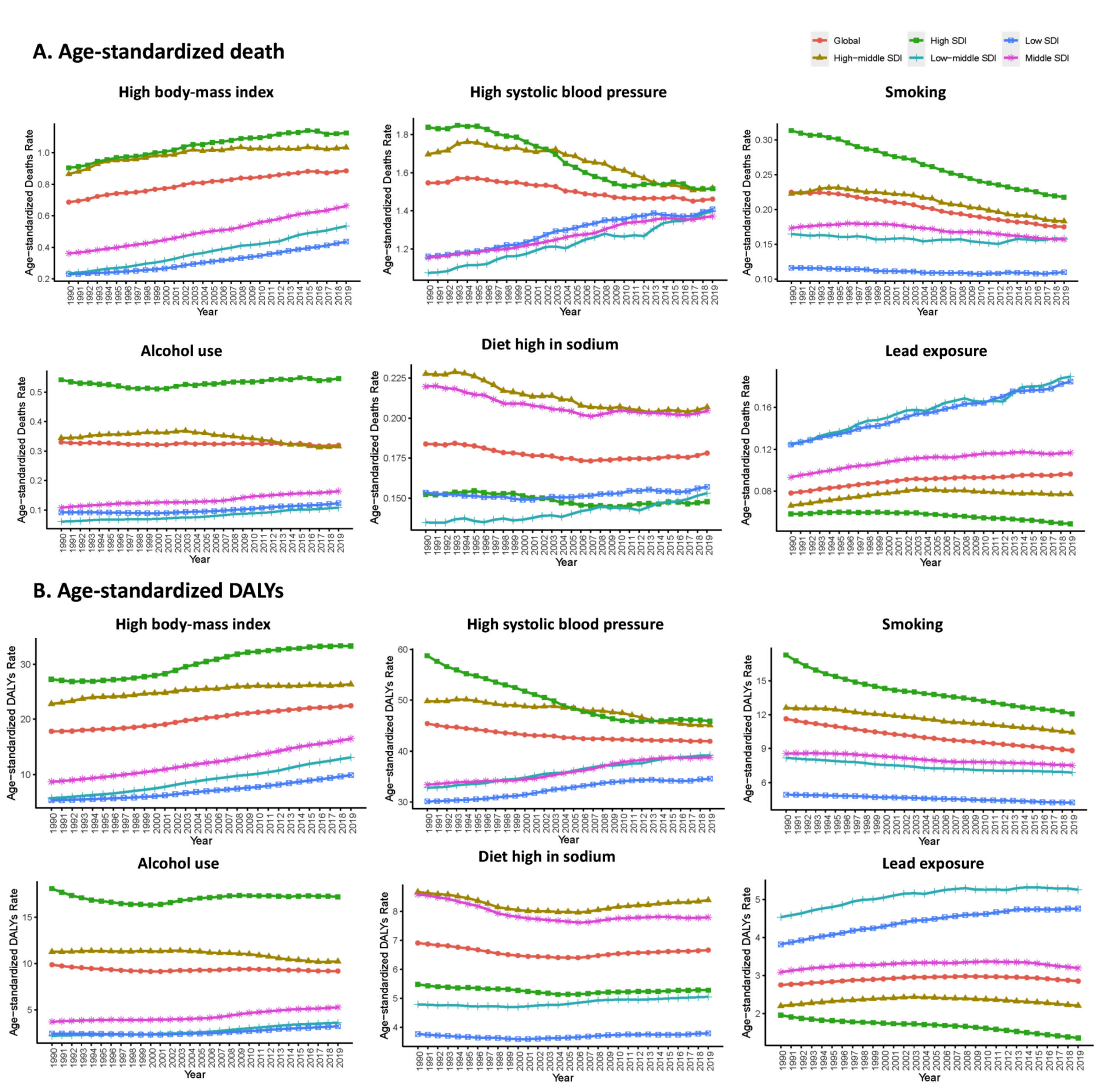
Major risk factors for age-standardised death of AF/AFL across SDI quintiles, 1990-2019. AF/AFL – atrial fibrillation/atrial flutter, SDI – sociodemographic index.

The distribution of risk variables differed across different SDI countries (**Figure 5**). The most prominent risk factors in high- and high-middle-income countries were a high body mass index (BMI), high systolic blood pressure, smoking, and alcohol consumption. In nations with a low- or low-middle-SDI, however, lead exposure was the most prevalent risk factor for AF/AFL mortality, with an upward trend. High blood pressure-related AF/AFL mortality decreased in high- and high-middle-SDI countries, but increased in low- and low-middle-SDI nations, while the incidence of AF/AFL deaths related to a high BMI increased across all SDI countries. In countries with a high or high-middle SDI, AF/AFL mortality was attributable to smoking. With the improvement of the level of socioeconomic development and medical care, the disease burden of young people began to decline, and thus more people lived to old age, further promoting the transfer of the disease burden to the elderly population. This trend increased with time, but remained stable in countries with a low or low-middle SDI. High-middle- and middle-SDI countries had the highest prevalence of AF/AFL mortality due to a high-sodium diet, while high, low, and low-middle SDI countries retained a lower incidence. We saw similar trends in the DALYs attributed to AF/AFL across SDI regions over time (**Figure 5**, Panel B). Generally, men and women had comparable trends in risk factors for AF/AFL-related deaths and DALYs across SDI quintiles (Figure S5 in the **Online Supplementary Document**). High BMI and high systolic blood pressure accounted for a greater proportion of AF/AFL deaths among women across all SDI quintiles over time, except in countries with a high SDI. Across all SDI quintiles, men experienced an increase in AF/AFL deaths attributable to smoking and alcohol use over time, and a greater percentage contribution of a high-sodium diet to age-standardised AF/AFL mortality in middle-SDI nations. Meanwhile, lead exposure-related mortality was comparable for men and women. Meanwhile, AF/AFL DALYs attributable to smoking, alcohol use, and lead exposure were more prevalent among men across SDI quintiles over time (Figure S5, Panel B in the **Online Supplementary Document**). However, men with a high BMI and high systolic blood pressure suffered greater AF/AFL DALYs in countries with a high SDI compared to those with other SDI quintiles. Sodium-rich diets resulted in greater DALYs for men in high-middle-SDI nations.

## DISCUSSION

We found that AF/AFL remains a serious health burden worldwide. Although the APC model estimated that the global net drift mortality of AF/AFL decreased, there was a rising tendency of net drift mortality in developing countries and areas. We observed a significant increase in all-age mortality and DALYs, an inverted U-shape across SDI quintiles in the percentage change of mortality and DALYs, an increase in the risk by age, and a shift in death and DALYs toward the older (>80) age group. From 1990 to 2019, Uzbekistan had the largest net drift of mortality and DALYs among all countries. High BMI, high blood pressure, smoking, alcohol consumption, and lead exposure were more prevalent in developed countries and regions.

Between 1990 and 2019, the global population increased by 45%, while the total number of AF/AFL deaths increased by 169%. The local drift analysis we performed showed that the increase in AF/AFL mortality was primarily attributable to the all-age upward trend in low-SDI nations and the older population in high- and high-middle-SDI countries. As aging has become a major social issue in developed countries [13], atrial fibrillation-related mortality, which increases with age, has become one of the most important public health crises in Western countries. In the past three decades, the use of anticoagulants and the promotion of catheter ablation techniques have reduced AF/AFL-related mortality and disability [14,15]. Although the incidence of AF/AFL is increasing, the related mortality is decreasing among well-treated individuals aged <80 years, with a similar pattern in AF/AFL mortality rates in developed nations. However, the rising prevalence of AF/AFL and lack of adequate treatment have contributed to an increase in related mortality and DALYs in low-SDI countries and regions [16,17]. Differences in population age composition (developed countries have longer life expectancies, and older people account for a greater proportion), socioeconomic level, education, and health development contribute to regional and national differences in atrial fibrillation-related deaths and DALYs.

The shift in AF/AFL mortality and DALYs to the older group was one of our key findings. According to the APC model analysis, it was most prominent in high- and middle-SDI nations and areas, which is rarely reported in other studies [18,19]. There are two possible explanations for this phenomenon. Although AF/AFL is an aging-related disease. the proportion of older people in developing countries, such as India, where the average life expectancy is 68.3 years, is low. The average life expectancy in 2020 in Japan, for example, was 83.7 years [20]. People in low-SDI countries are more likely to die or become disabled from other diseases (diseases with an earlier age of onset, such as ischemic heart disease). Second, AF/AFL awareness and diagnostic rates are low in countries with a low SDI, resulting in bias in data [21]. Consistent with previous studies, the number of AF/AFL-related deaths and DALYs was higher among women than among men in 2019. Nonetheless, based on the APC model, we found a greater downward trend or smaller upward trend of AF/AFL-related deaths among women across SDI quintiles, except for low- and high-SDI countries, indicating that the focus on women's health has been effective over the past few decades. In a national study in Singapore, men had 17% increased odds of mortality compared to women one year after a diagnosis of AF/AFL [22]. The high level of inclusive medical care in countries with a high SDI and the low-quality medical care in countries with a low SDI contributed to sex-homogeneous treatment results.

There were also large disparities in AF/AFL deaths or DALYs within the same SDI levels. For example, Japan’s AF/AFL death and DALY rates were more similar to those of Italy due to its rapidly aging population, diet regimens, and climate. SDI is an indicator assessed as a composite of income per capita, average years of schooling, and the fertility rate among women aged <25 years, and is more associated with economic level. The clinical outcome of atrial fibrillation is impacted by numerous factors, only one of which is the SDI, so a customised evaluation approach is needed to assess and classify the global disease burden.

Previous studies have analysed the attributable risk factors for AF/AFL deaths and DALYs based on GBD data [18,19]. In this study, hypertension, obesity, smoking and alcohol use were also highly related to AF/AFL mortality and DALYs, particularly in high- and high-middle-SDI countries. The subgroup analysis based on sex showed that hypertension and obesity should receive more attention in the female population, while smoking and drinking should receive more attention in the male population. Importantly, we also found that AF/AFL mortality and DALYs attributable to lead exposure are on the rise in low- and low-middle-SDI countries for both male and female populations. Previous studies determined that lead exposure was strongly associated with hypertension [23], acute ischemic stroke [24], and heart failure [25], and that reducing lead exposure can result in substantial advantages for the adult population [26]. Lead exposure should be a prioritised topic, especially in nations and regions with a low or low-middle SDI.

### Limitations

This study has some limitations. First, death rates for paroxysmal and chronic atrial fibrillation varied substantially, but no classification was provided by the data. We also lacked sufficient data on anticoagulation, anti-arrhythmia medication therapy, and radiofrequency for detailed analyses. Third, the risk score classification plays a crucial role in predicting clinical outcomes for atrial fibrillation, which was unavailable in the GBD data, as were the life expectancy data for each country, which may have helped better determine the effects of life expectancy on the disease burden. Nevertheless, large data analytics across countries, SDIs, and age groups can still show the worldwide patterns of AF/AFL mortality and sex differences and guide future health policy interventions.

## CONCLUSIONS

We found that the burden of AF/AFL in 2019 and its temporal evolution from 1990 to 2019 varied significantly by SDI quintile, sex, geographic region, and country. Even though global net drift mortality and the DALYs of AF/AFL decreased, there was an upward trend in the net drift in the elderly population in high-SDI nations compared to the overall population in low- and low-middle-SDI countries. Special attention should be given to hypertension, obesity, smoking, and alcohol consumption in high- and high-middle-SDI countries, while lead exposure should be prioritised in developing nations. The increasing overall awareness and diagnostic opportunities are a major force in detecting AF/AFL, and readiness and treatment opportunities are also critical in the achievement of national goals. Health authorities and policymakers must consider improving the allocation of resources, increasing access to health care resources, controlling variable risk factors, and urgently improving efforts to curb the growth of the disease burden.

## Additional material


Online Supplementary Document
