# Supplementary material for: Global, regional, and national burdens of atrial fibrillation/flutter from 1990 to 2019: An age-period-cohort analysis using the Global Burden of Disease 2019 study
Source: J Glob Health. 2023 Nov 22;13:04154. doi: 10.7189/jogh.13.04154 (PMC10662782; doi:10.7189/jogh.13.04154)
Supplement: Online Supplementary Document [file jogh-13-04154-s001.pdf]

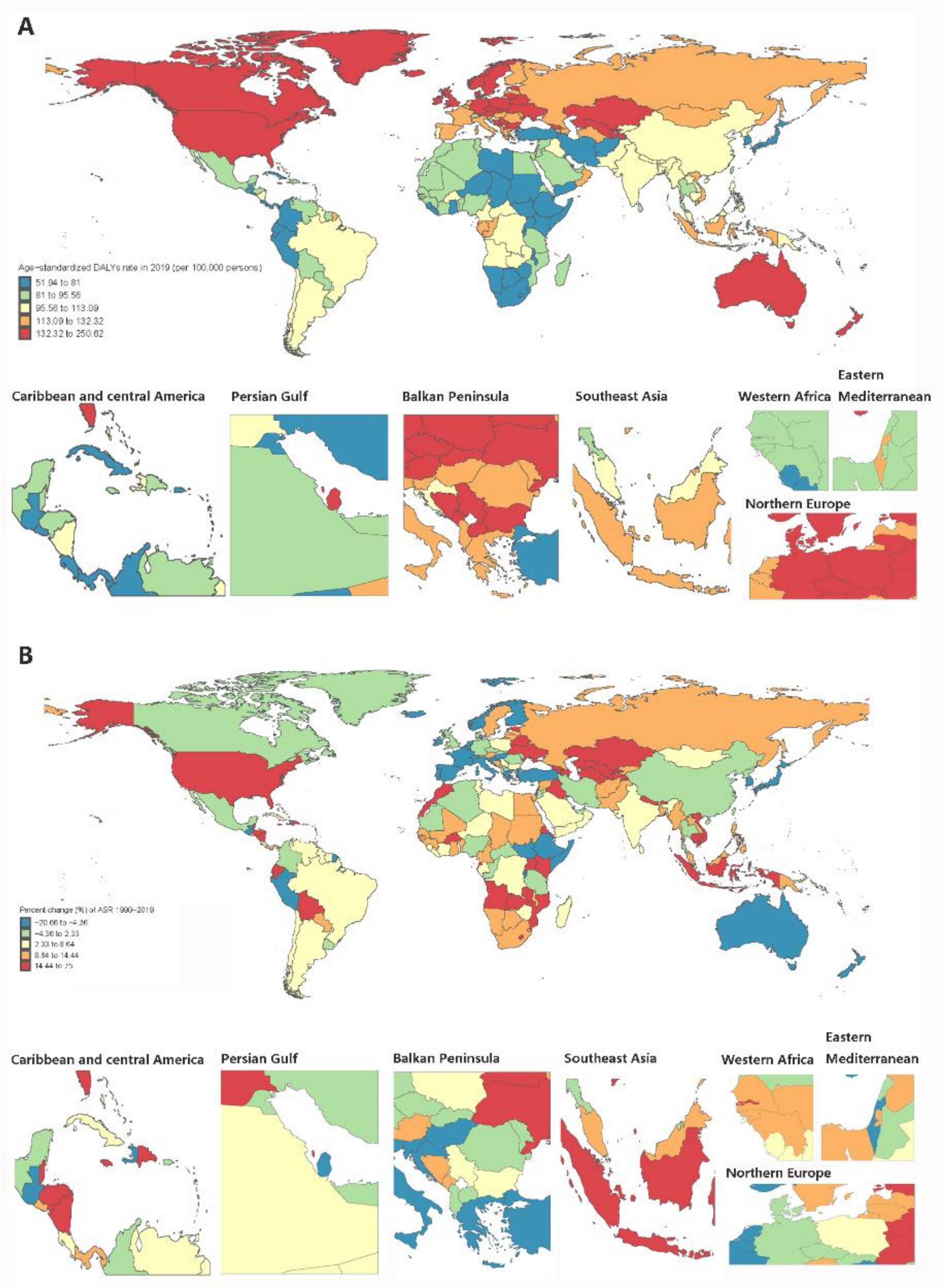

Figure S1 Age-standardized DALYs (A), and percentage of DALY (B) rates of atrial fibrillation /atrial flutter across 204 countries and territories from 1990 to 2019. DALY, disability-adjusted life-years.

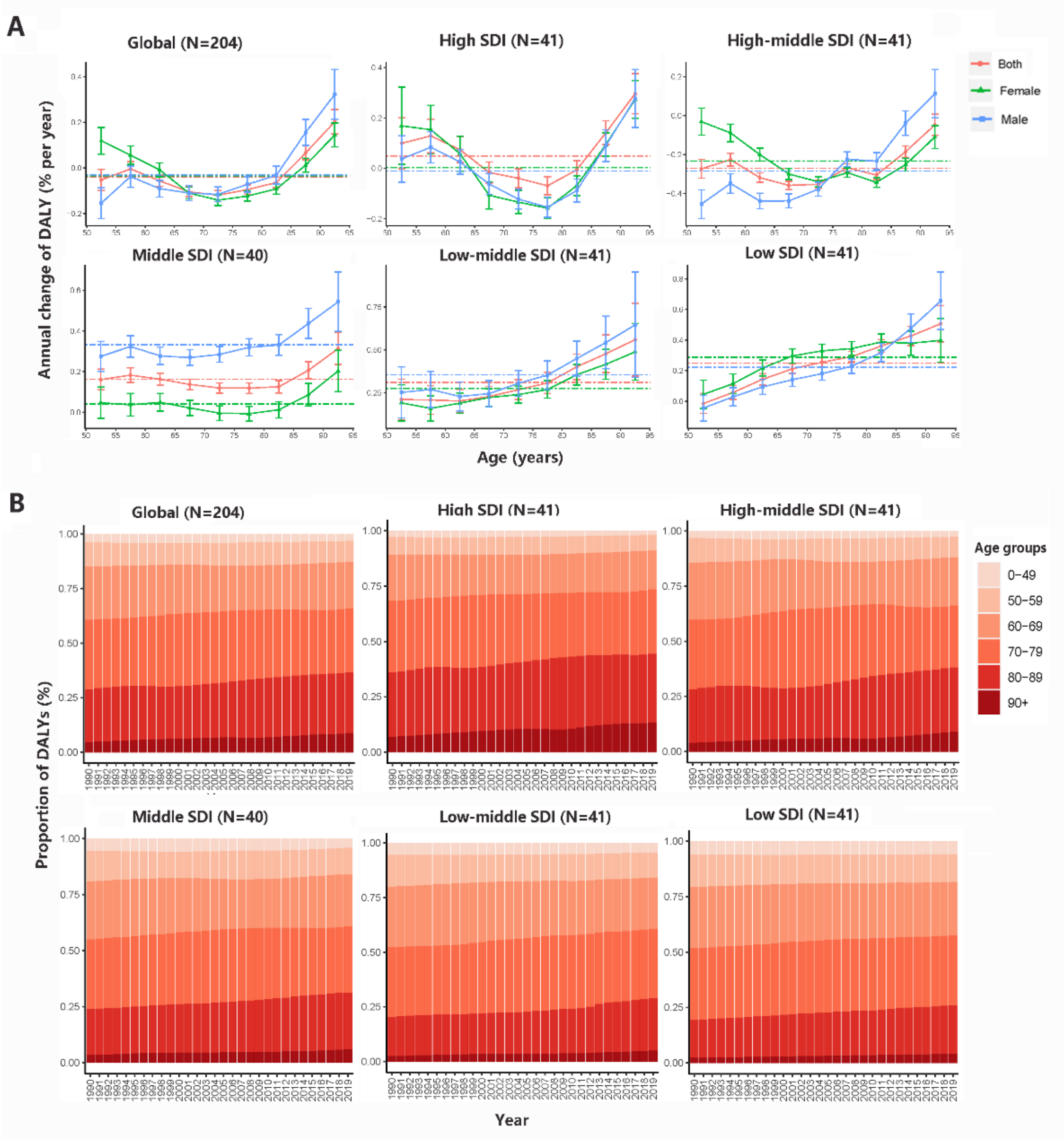

Figure S2 Local drifts of AF/AFL DALYs and age distribution of DALYs from AF/AFL by SDI quintiles, 1990-2019. DALY, disability-adjusted life-years; SDI, sociodemographic index.

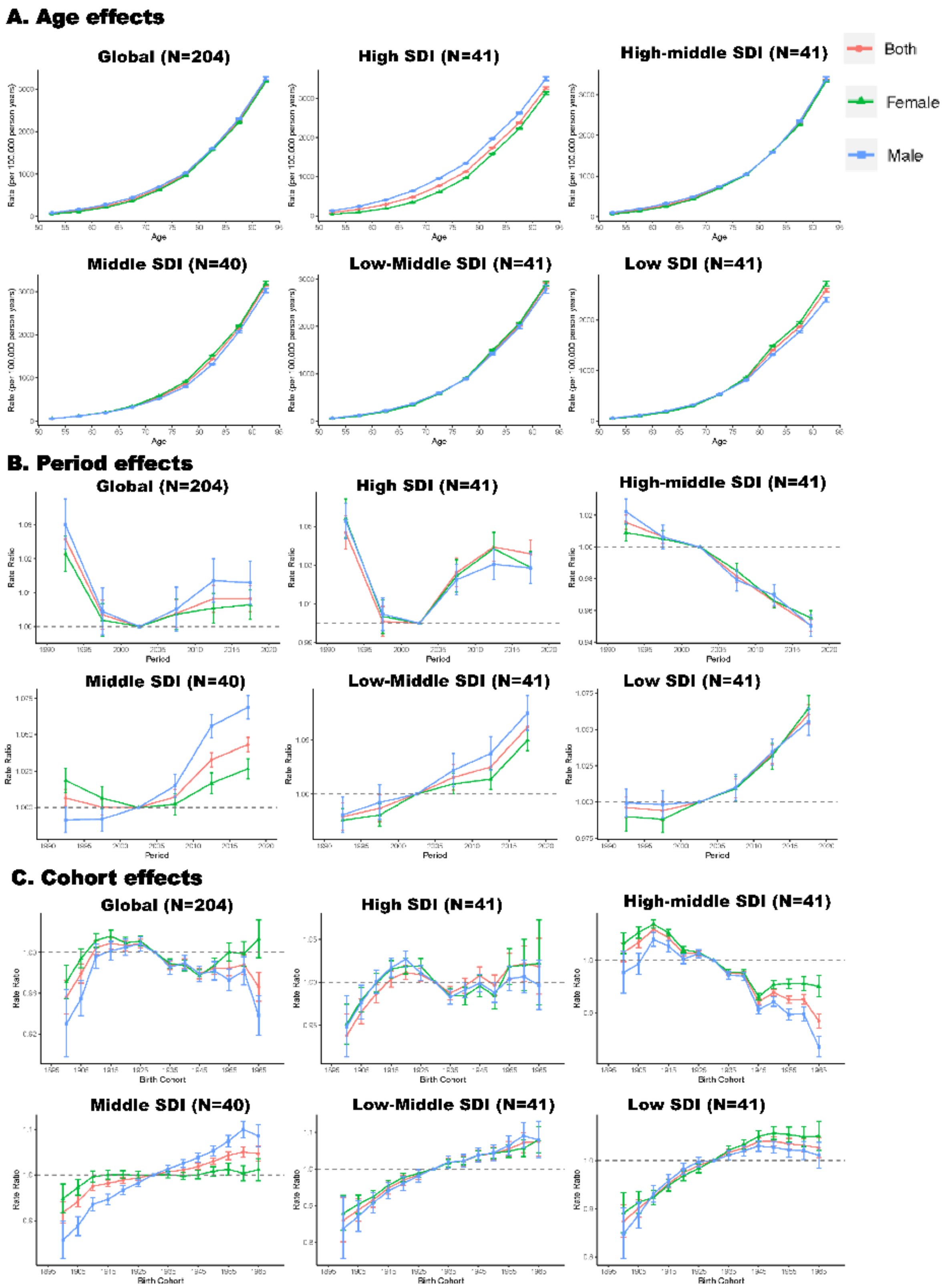

Figure S3 Age, period and cohort effects on atrial fibrillation/ atrial flutter DALYs by SDI quintiles. DALY, disability-adjusted life-years; SDI, sociodemographic index.

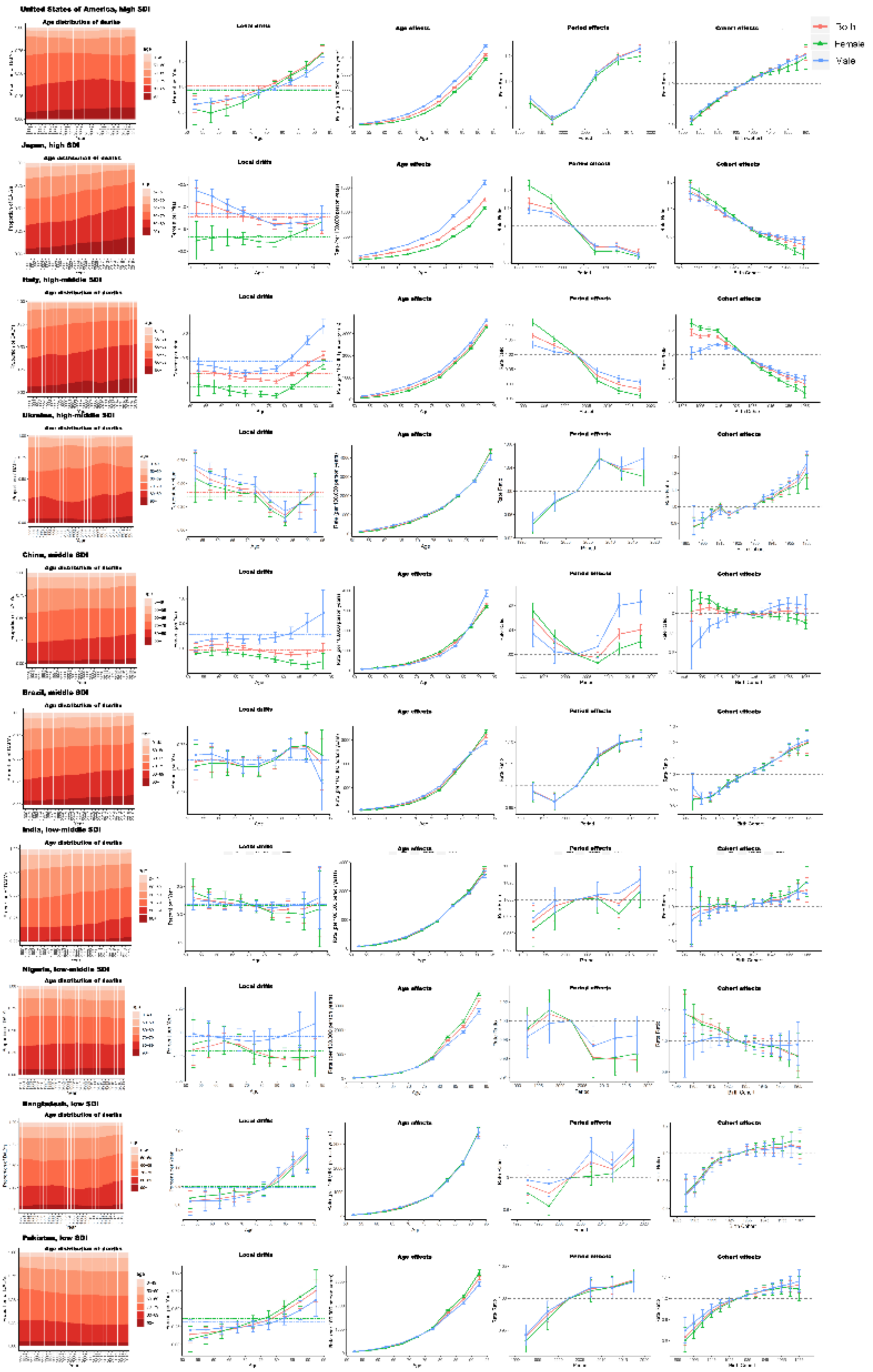

Figure S4 Age distribution of atrial fibrillation/atrial flutter death and age-period-cohort effects on exemplar countries across SDI quintiles. SDI, sociodemographic

index.

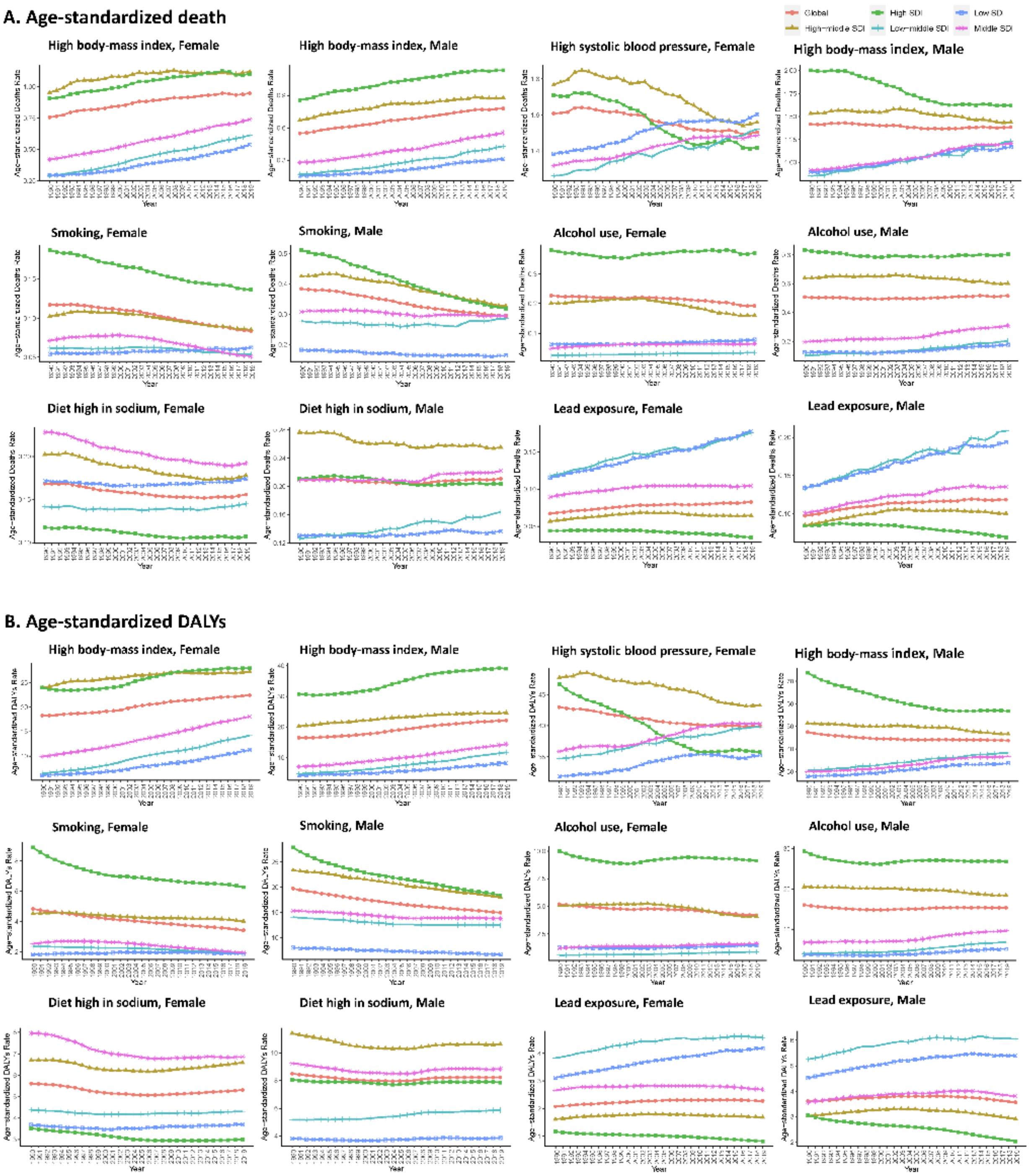

Figure S5 **Major risk factors to age-standardized DALYs of atrial fibrillation/atrial flutter across SDI quintiles, 1990–2019.** DALY, disability-adjusted life-years; SDI, sociodemographic index.

Table S1. Trends in AF/AFL DALYs across Socio-demographic Index quintiles, 1990-2019

	Global		High SDI		High-middle SDI		Middle SDI		Low-middle SDI		Low SDI	
	1990	2019	1990	2019	1990	2019	1990	2019	1990	2019	1990	2019
**DALYs**												
Number*, n× 1,000	3787.84(2961.19,4832.67)	8393.64(6693.98,10541.46)	1361.23(1075.4,1754.75)	2517.23(2012.61,3144.01)	1108.7(863.01,1438.27)	2214.11(1734.63,2823.35)	745.66(576.3,952.66)	2113.47(1653.61,2694.93)	421.27(319.7,540.87)	1173.89(929.51,1468.08)	149.12(112.1,190.13)	370.88(291.5,456.05)
Percentage of global,%	100	100	35.94	29.99	29.27	26.38	19.69	25.18	11.12	13.99	3.94	4.42
Percent change of DALYs, 1990–2019,%	122(112,132)		85 (74,95)		100(91,108)		183(165,203)		179(154,208)		149 (126,178)	
**All-age DALY rate**												
Rate per 100,000	70.8(55.35,90.33)	108.48(86.51,136.24)	165.6(130.83,213.47)	248.4(198.6,310.25)	96.37(75.02,125.02)	154.79(121.27,197.38)	43.43(33.57,55.49)	88.19(69,112.45)	37.29(28.3,47.88)	66.55(52.69,83.23)	28.24(21.22,36)	32.86(25.83,40.41)
Percent change of rate 1990–2019,%	53(46,60)		50(41,58)		61(53,67)		103(89,117)		78(63,97)		16(6,30)	
**Age-standardized DALY rate**												

Rate per 100,000	110(87.66,139.16)	107.13(86.18,133.73)	128.62(101.73,165.21)	122.64(97.3,153.57)	116.99(92.57,149.81)	110.21(86.73,140.14)	93.38(74.4,116.56)	97.9(78.07,122.72)	91.5(71.71,114.77)	101.01(81.22,123.92)	84.28(63.03,105.84)	91.91(71.89,111.57)
Percent change of rate 1990–2019, %	-3(-7,1)		-5(-10,-1)		-6(-10,-2)		5(-2,12)		10(0,22)		9(-2,23)	
**APC model estimates**												
Net drift of DALY, y,% per year	-0.04(-0.05, -0.02)		0.05(0.02,0.08)		-0.27(-0.28,-0.26)		0.16(0.14,0.18)		0.31(0.27,0.35)		0.25(0.23,0.27)	

Notes: All-age mortality=crude mortality rate. Age-standardized mortality rate is computed by direct standardization with global standard population in GBD 2019. * Parentheses for all GBD health estimate indicate 95% uncertainty intervals; parentheses for net drift indicate 95% confidence intervals; Net drifts are estimates derived from the age-period-cohort model and denotes overall annual percentage change in mortality, which captures the contribution of the effects from calendar time and successive birth cohorts; AF/AFL, atrial fibrillation/ atrial flutter; SDI=Socio-demographic Index; APC=age-period-cohort. DALY, disability-adjusted life-years.

Table S2 **Number of deaths, all-age mortality, age-standardized mortality and net drift of mortality distribution in 204 countries and territories.**

SDI groups	Countries/Regions	Number of deaths in 2019	All-age mortality rate, per 100,000 in 2019	Age-standardized mortality rate in 2019	Net drift of mortality y, % per year
High SDI	Denmark	907.05(729.29,1072.41)	15.63(12.57,18.48)	6.73(5.42,7.98)	-0.18(-0.93,0.59)
	Puerto Rico	369.91(263.83,468.68)	10.5(7.49,13.31)	3.88(2.81,4.93)	-0.17(-1.32,0.99)
	Switzerland	795.55(617.87,953.76)	9.07(7.04,10.87)	3.38(2.66,4.05)	-0.62(-1.55,0.31)
	Russian Federation	9962.22(8165.77,13099.37)	6.79(5.57,8.93)	4.27(3.5,5.65)	0.28(0.1,0.46)
	Germany	18195.7(14793.28,22483.09)	21.43(17.42,26.48)	7.42(6.07,9.21)	0.27(-0.03,0.56)
	Finland	750.37(484.08,864.94)	13.56(8.75,15.63)	4.77(3.08,5.48)	-0.5(-1.41,0.41)
	France	9252.63(6808.1,10772.53)	13.98(10.28,16.27)	4.59(3.44,5.39)	-0.71(-1.01,-0.42)
	Czechia	940.34(719.25,1145.34)	8.83(6.76,10.76)	4.17(3.17,5.09)	-0.39(-1.04,0.27)
	United Kingdom	9031.08(6630.04,10127.33)	13.44(9.86,15.07)	5.86(4.33,6.56)	-0.21(-0.47,0.05)
	San Marino	4.67(3.33,6.17)	14.1(10.07,18.65)	5.24(3.71,6.99)	0.33(-13.36,16.18)
	Brunei Darussalam	10.42(9.09,11.92)	2.38(2.08,2.73)	8.1(6.99,9.22)	-0.21(-4.65,4.45)
	Kuwait	38.29(29.2,53.21)	0.86(0.66,1.2)	2.21(1.67,3.04)	0.04(-3.54,3.75)
	Ireland	483.11(348.02,552.57)	9.84(7.09,11.25)	6.08(4.38,6.95)	-1.26(-2.27,-0.24)
	Republic of Korea	2022.03(1640.38,2301.69)	3.79(3.07,4.31)	2.57(2.05,2.94)	-1.03(-1.48,-0.59)
	Taiwan (Province of China)	1399.08(1069.94,1946.13)	5.92(4.53,8.24)	3.32(2.54,4.61)	-0.03(-0.69,0.62)
	Luxembourg	69.07(53.61,86.8)	11.17(8.67,14.03)	5.69(4.45,7.19)	-0.99(-3.95,2.06)
	Singapore	134.93(99,163.32)	2.38(1.75,2.88)	1.97(1.44,2.37)	-1.76(-3.35,-0.15)
	Slovenia	236.17(164.95,304.24)	11.39(7.95,14.67)	4.37(3.04,5.66)	-0.65(-2.07,0.79)
	United States of America	33210.82(26672.81,40280.22)	10.13(8.13,12.28)	5.08(4.12,6.16)	0.6(0.49,0.71)
	Australia	3422.81(2739.7,4171.64)	13.93(11.15,16.98)	6.79(5.41,8.24)	-0.84(-1.3,-0.38)
	Iceland	41.08(32.03,52.23)	11.91(9.29,15.14)	5.78(4.58,7.4)	-0.84(-4.65,3.13)

	Canada	3227.65(2504.81,3892.3)	8.84(6.86,10.66)	3.97(3.09,4.84)	-0.91(-1.3,-0.53)
	Japan	13139.9(9794.8,18258.52)	10.28(7.66,14.29)	2.39(1.83,3.27)	-0.84(-1.04,-0.63)
	Slovakia	456.54(365.82,557.04)	8.4(6.73,10.24)	5.17(4.13,6.3)	-0.24(-1.06,0.59)
	New Zealand	658.79(518.98,793.73)	14.65(11.54,17.66)	7.21(5.71,8.71)	-0.56(-1.5,0.38)
	Estonia	148.91(116.2,210.11)	11.35(8.85,16.01)	4.58(3.59,6.43)	0.43(-1.26,2.15)
	Belgium	1770.24(1431.41,2231.31)	15.5(12.54,19.54)	5.71(4.61,7.2)	-0.41(-1.02,0.2)
	Qatar	21.19(15.46,28.28)	0.74(0.54,0.99)	12.12(9.13,16.76)	-1.29(-4.75,2.3)
	Saudi Arabia	300.84(235.69,384.02)	0.84(0.66,1.07)	4.29(3.35,5.29)	-0.16(-1.04,0.72)
	Latvia	196.95(158.92,245.19)	10.28(8.3,12.8)	4.11(3.32,5.12)	0.1(-1.35,1.57)
	Austria	1453.34(1132.72,1655.48)	16.3(12.7,18.57)	6.27(4.89,7.12)	0.13(-0.56,0.82)
	Andorra	9.7(6.51,12.96)	11.67(7.84,15.6)	5.78(3.83,7.76)	-0.01(-7.62,8.23)
	Netherlands	2064.85(1673.09,2677.49)	12.04(9.75,15.61)	5.27(4.27,6.83)	-0.97(-1.49,-0.45)
	United Arab Emirates	38.22(20.53,71.81)	0.41(0.22,0.78)	4.12(2.25,7.16)	-0.22(-3.47,3.14)
	Lithuania	291.28(232.73,360.81)	10.42(8.33,12.91)	4.18(3.36,5.19)	0.04(-1.15,1.25)
	Bermuda	6.98(5.44,8.95)	10.89(8.49,13.98)	4.69(3.67,6)	-1.65(-9.87,7.32)
	Cyprus	127.22(108.27,144.06)	9.69(8.24,10.97)	8.14(6.79,9.25)	-1.46(-3.52,0.65)
	Guam	5.79(4.7,6.97)	3.39(2.75,4.09)	3.1(2.52,3.72)	-2.13(-8.07,4.19)
	Norway	824.09(542.18,935.91)	15.41(10.14,17.5)	6.76(4.49,7.69)	-0.83(-1.73,0.08)
	Sweden	2450.54(1110.45,2942.7)	23.97(10.86,28.79)	8.63(4,10.32)	0.61(-0.12,1.33)
	Monaco	6.51(5.02,7.82)	17.33(13.36,20.81)	4.96(3.87,5.96)	-0.14(-10.13,10.97)
High-middle SDI	Croatia	318.95(249.6,439.02)	7.51(5.88,10.34)	3.39(2.65,4.62)	-0.75(-1.83,0.33)
	Ukraine	4434.82(3631.52,5949.3)	10.07(8.25,13.51)	5.9(4.79,7.92)	0.36(0.11,0.61)
	Hungary	897.52(716.57,1143.89)	9.28(7.41,11.82)	4.21(3.36,5.37)	-0.48(-1.11,0.16)
	Argentina	2906.38(2404.95,3729.81)	6.44(5.33,8.27)	5.14(4.25,6.59)	-0.01(-0.43,0.41)
	North Macedonia	161.92(133.39,195.64)	7.52(6.2,9.09)	8.22(6.78,9.93)	-0.12(-1.48,1.25)

	Kazakhstan	716.7(559.43,1286.07)	3.9(3.04,6.99)	6.64(5.18,11.75)	0.48(-0.11,1.08)
	Romania	1473.46(932.74,1773.2)	7.66(4.85,9.22)	3.61(2.31,4.34)	-0.27(-0.81,0.26)
	Chile	1410.6(1164.86,2052.4)	7.75(6.4,11.28)	5.97(4.91,8.69)	0.3(-0.28,0.88)
	Poland	4089.5(3114.3,4974.66)	10.64(8.1,12.94)	5.33(4.05,6.47)	0.3(-0.01,0.61)
	Montenegro	120.89(96.99,160.4)	19.49(15.63,25.86)	14.38(11.31,19.38)	0.88(-0.67,2.44)
	Greece	1742.41(1360.24,3019.94)	16.86(13.16,29.21)	5.23(4.12,8.96)	-0.51(-1.08,0.06)
	Malaysia	720.52(561.4,931.99)	2.3(1.79,2.98)	4.05(3.12,5.31)	0.85(0.18,1.52)
	Lebanon	196.42(124.04,255.14)	3.79(2.4,4.93)	4.07(2.57,5.27)	-0.26(-1.7,1.21)
	Serbia	803.28(649.62,1110.6)	9.18(7.43,12.7)	6.28(5.08,8.77)	0.03(-0.6,0.67)
	Israel	641.82(520.7,862.78)	6.89(5.59,9.27)	4.8(3.91,6.44)	-1.63(-2.54,-0.72)
	Bulgaria	931.3(720.34,1447.57)	13.43(10.39,20.87)	6.41(4.99,9.99)	0.75(0.2,1.31)
	Italy	10569.32(8459.39,14448.73)	17.52(14.03,23.96)	5.23(4.22,7.07)	-1.01(-1.27,-0.75)
	Bosnia and Herzegovina	294.76(231.66,388.22)	8.93(7.02,11.76)	5.75(4.53,7.48)	0.57(-0.39,1.54)
	Jordan	138.66(112.79,163.43)	1.19(0.97,1.4)	4.39(3.44,5.19)	-0.76(-2.46,0.96)
	Belarus	751.68(585.84,964.56)	7.91(6.17,10.15)	4.5(3.51,5.78)	0.72(0.02,1.43)
	Portugal	1253.39(1012.87,1860.88)	11.77(9.51,17.47)	3.97(3.23,5.84)	-1.17(-1.86,-0.48)
	Libya	103.91(68.97,142.43)	1.54(1.02,2.11)	2.66(1.78,3.63)	0.62(-1.33,2.61)
	Turkey	2416.1(1885.67,3072.99)	2.97(2.32,3.78)	3.15(2.43,3.97)	-1.52(-1.9,-1.14)
	Spain	7378.09(5887.35,9277.72)	16.03(12.79,20.16)	5.41(4.39,6.83)	-0.8(-1.12,-0.47)
	Dominica	7.15(5.92,8.64)	10.41(8.62,12.59)	7.9(6.54,9.56)	0.64(-6.18,7.95)
	Saint Kitts and Nevis	4.24(3.59,5.34)	7.12(6.04,8.98)	10.06(8.48,12.53)	-0.37(-7.79,7.64)
	United States Virgin Islands	10.73(9.07,12.91)	10.31(8.72,12.41)	7.46(6.24,8.97)	1.26(-4.03,6.85)
	Antigua and Barbuda	6.07(5.06,7.72)	6.86(5.72,8.72)	7.8(6.45,9.95)	0.37(-6.85,8.15)
	Barbados	27.55(22.09,34.79)	9.25(7.42,11.68)	5.71(4.59,7.19)	0.14(-3.72,4.16)
	Trinidad and Tobago	75.26(53.29,96.7)	5.42(3.84,6.97)	4.46(3.16,5.73)	0.35(-1.72,2.47)

	Bahamas	19.23(15.42,25.23)	5.1(4.09,6.69)	6.32(5.06,8.22)	0.31(-3.57,4.34)
	Malta	45.14(35.86,55.45)	10.28(8.16,12.62)	4.24(3.38,5.21)	-0.89(-4.49,2.85)
	Seychelles	3.49(2.44,4.6)	3.41(2.39,4.5)	4.17(2.87,5.56)	0.05(-8.61,9.54)
	Cook Islands	1.07(0.86,1.29)	5.95(4.78,7.18)	4.79(3.85,5.82)	-0.34(-13.75,15.16)
	American Samoa	1.75(1.4,2.17)	3.15(2.53,3.92)	4.92(3.92,6.14)	0.1(-10.05,11.39)
	Bahrain	35.29(25.84,43.79)	2.45(1.79,3.04)	10.65(8.28,12.78)	3.47(-0.61,7.72)
	Oman	41.08(32,49.44)	0.9(0.7,1.08)	8.38(5.14,10.31)	-0.56(-3.12,2.06)
	Greenland	4.48(3.57,5.29)	7.98(6.35,9.41)	9.35(7.48,10.94)	-0.35(-6.67,6.4)
	Niue	0.1(0.08,0.13)	6.23(4.62,7.83)	5.05(3.75,6.36)	0(-32.41,47.96)
	Northern Mariana Islands	1.96(1.64,2.26)	4.61(3.87,5.32)	6.73(5.63,7.83)	0.67(-10.03,12.64)
	Palau	0.4(0.32,0.49)	2.22(1.77,2.73)	3.2(2.53,3.87)	-0.17(-18.32,22.01)
Middle SDI	Costa Rica	203.87(143.98,259.82)	4.32(3.05,5.51)	3.82(2.69,4.88)	0.1(-1.43,1.66)
	Iran (Islamic Republic of)	1766.41(1509.85,2003.07)	2.1(1.79,2.38)	3.16(2.68,3.59)	0.17(-0.33,0.67)
	Armenia	162.48(132.25,218.99)	5.38(4.38,7.25)	4.35(3.53,5.98)	0.83(-0.68,2.36)
	Azerbaijan	226.13(188.77,261.61)	2.2(1.84,2.55)	5.69(4.57,6.67)	1.35(0.25,2.46)
	Iraq	630.49(480.69,1022.37)	1.5(1.14,2.43)	4.43(3.37,7.52)	0.75(0.05,1.44)
	Sri Lanka	599.09(453.59,758.06)	2.74(2.08,3.47)	3.4(2.53,4.3)	0.77(-0.05,1.6)
	China	51747.82(43604.73,60138.02)	3.64(3.07,4.23)	3.84(3.2,4.45)	-0.67(-0.78,-0.56)
	Georgia	421.47(332.46,505.1)	11.5(9.07,13.78)	6.03(4.74,7.17)	2.37(1.47,3.28)
	Thailand	3482.78(2557.84,4434.23)	4.97(3.65,6.32)	3.63(2.66,4.62)	-0.81(-1.21,-0.42)
	Uruguay	322.25(216.11,367.56)	9.38(6.29,10.7)	4.62(3.13,5.27)	-0.14(-1.44,1.18)
	South Africa	1205.85(1038.92,1349.53)	2.17(1.87,2.43)	3.96(3.37,4.42)	0.67(0.19,1.16)
	Mexico	5227.84(4191.07,7001.67)	4.18(3.35,5.6)	5.23(4.17,7.05)	0.05(-0.21,0.32)
	Jamaica	197.23(149.6,268.12)	7.02(5.32,9.54)	5.3(4.03,7.38)	0.57(-0.86,2.03)
	Indonesia	4991.79(3897.51,6555.52)	1.92(1.5,2.53)	4.33(3.37,5.65)	0.96(0.73,1.19)

Egypt	1311.05(852.93,1799.86)	1.32(0.86,1.82)	3.93(2.51,5.38)	0.66(0.25,1.08)
Paraguay	239.94(180.38,310.61)	3.46(2.6,4.48)	4.69(3.54,6.09)	0.77(-0.45,2.02)
Algeria	966.89(739.66,1230.25)	2.31(1.77,2.94)	4.87(3.67,6.11)	-0.2(-0.81,0.42)
Panama	180.17(131.3,240.76)	4.33(3.16,5.79)	3.99(2.93,5.39)	0.11(-1.55,1.8)
Peru	1176.69(881.68,1537.53)	3.46(2.59,4.52)	3.52(2.65,4.62)	-0.74(-1.31,-0.16)
Albania	157.39(119.04,198.3)	5.79(4.38,7.29)	3.97(3.01,4.98)	-0.09(-1.65,1.5)
Uzbekistan	503.53(419.46,627.96)	1.5(1.25,1.86)	8.6(6.78,10.05)	4.21(3.51,4.9)
Brazil	10811.37(8636.51,12800.84)	4.99(3.99,5.91)	5.04(4.01,5.97)	0.3(0.11,0.48)
Ecuador	643.67(515.95,792.45)	3.66(2.93,4.51)	5.57(4.47,6.82)	0.93(0.08,1.79)
Cuba	926.03(715.99,1222.07)	8.15(6.3,10.76)	4.27(3.32,5.68)	0.09(-0.6,0.78)
Colombia	2128.15(1528.08,2833.96)	4.45(3.2,5.93)	3.64(2.64,4.76)	-0.17(-0.65,0.32)
Tunisia	383.37(279.69,502.41)	3.31(2.42,4.34)	3.9(2.83,5.07)	0.47(-0.53,1.47)
Republic of Moldova	195.93(156.45,246.88)	5.31(4.24,6.69)	3.41(2.72,4.29)	-0.58(-1.83,0.69)
Gabon	46.66(33.14,61.82)	2.67(1.89,3.53)	6.92(4.87,9.21)	0.05(-2,2.14)
Grenada	4.83(4.12,6.38)	4.68(3.99,6.18)	5.94(5.01,7.87)	0.04(-6.59,7.15)
Tokelau	0.05(0.04,0.07)	3.89(3.07,4.8)	4.87(3.86,6)	0.12(-41.53,71.44)
Botswana	27.55(19.75,37.32)	1.18(0.84,1.6)	3.8(2.71,5.21)	-0.01(-2.69,2.75)
Saint Lucia	14.21(11.55,17.88)	8.14(6.61,10.24)	7.55(6.1,9.43)	-0.87(-6.07,4.61)
Fiji	22.52(18.35,27.72)	2.47(2.01,3.04)	5.22(4.3,6.32)	0.71(-2.13,3.64)
Saint Vincent and the Grenadines	7.6(6.32,9.89)	6.71(5.59,8.74)	7.02(5.84,9.12)	0.09(-6.28,6.88)
Suriname	26.19(20.93,31.61)	4.55(3.63,5.49)	5.08(4.06,6.16)	0.19(-3.19,3.69)
Turkmenistan	126.2(100.4,171.28)	2.48(1.98,3.37)	4.65(3.7,6.27)	1.17(-0.33,2.68)
Mauritius	53.98(42.68,70.69)	4.23(3.34,5.54)	3.65(2.88,4.76)	-0.2(-2.46,2.11)
Tonga	3.08(2.3,3.92)	3.01(2.25,3.83)	4.14(3.09,5.28)	0.46(-7.62,9.23)
Samoa	5.67(4.45,7.14)	2.68(2.1,3.38)	4.94(3.87,6.22)	-0.15(-5.69,5.7)

	Equatorial Guinea	19.56(12.48,29.34)	1.38(0.88,2.07)	6.96(4.49,10.58)	1.34(-2.54,5.38)
Low-middle SDI	Philippines	1571.2(1298.61,2070.43)	1.4(1.16,1.85)	3.25(2.68,4.25)	2.02(1.52,2.53)
	Ghana	330.18(271.47,403.67)	1.05(0.86,1.28)	3.93(3.19,4.77)	0.75(-0.38,1.9)
	Myanmar	1113.51(910.95,1384.48)	2.04(1.67,2.53)	3.56(2.88,4.4)	0.54(0.05,1.03)
	Sudan	399.51(298.6,515.96)	0.98(0.73,1.26)	3.19(2.35,4.14)	0.27(-0.53,1.08)
	Zambia	242.05(166.24,307.72)	1.33(0.91,1.69)	6.43(4.49,8.1)	1.62(0.54,2.72)
	Democratic People's Republic of Korea	918.76(732.09,1113.23)	3.5(2.79,4.24)	3.85(3.02,4.74)	-0.02(-0.57,0.52)
	Dominican Republic	437.39(343.1,539.97)	4.02(3.15,4.96)	5.52(4.32,6.81)	1.37(0.36,2.38)
	Guatemala	285.98(210.9,352.53)	1.61(1.19,1.98)	3.72(2.74,4.49)	-0.91(-1.97,0.16)
	El Salvador	294.36(219.17,364.39)	4.71(3.5,5.82)	4.14(3.09,5.14)	0.13(-1.16,1.44)
	Honduras	227.07(173.1,294.14)	2.31(1.76,3)	5.74(4.27,7.56)	1.91(0.56,3.27)
	Kyrgyzstan	115.77(88.74,134.11)	1.77(1.36,2.05)	3.67(2.78,4.26)	0.48(-1.08,2.07)
	Tajikistan	143.33(112.46,171.62)	1.51(1.18,1.81)	7.33(5.59,8.78)	1.5(0.14,2.88)
	India	29154.86(22579,37299.87)	2.1(1.62,2.68)	3.99(3.07,5.12)	0.31(0.13,0.49)
	Nicaragua	190.45(160.33,224.54)	2.93(2.46,3.45)	6.52(5.45,7.64)	0.98(-0.49,2.47)
	Viet Nam	3124.2(2391.35,4041.79)	3.24(2.48,4.19)	4.63(3.53,5.96)	0.45(0.11,0.79)
	Kenya	600.39(395.55,800.66)	1.2(0.79,1.59)	4.99(3.23,6.74)	1.16(0.47,1.86)
	Nigeria	2366.61(1778.66,2903.56)	1.1(0.83,1.35)	4.81(3.58,5.91)	-0.46(-0.84,-0.07)
	Morocco	874.38(664.61,1082.18)	2.43(1.85,3.01)	4.37(3.2,5.55)	0.85(0.22,1.48)
	Venezuela (Bolivarian Republic of)	1169.25(861.74,1510.97)	4.17(3.07,5.38)	4.39(3.26,5.69)	0.11(-0.5,0.72)
	Bolivia (Plurinational State of)	418.6(308.6,560.02)	3.48(2.57,4.66)	6.75(4.95,8.92)	0.41(-0.48,1.31)
	Cameroon	349.86(274.44,439.2)	1.2(0.94,1.51)	5.78(4.46,7.24)	0.54(-0.49,1.57)
	Syrian Arab Republic	296.78(218.34,385.21)	2.05(1.51,2.66)	4.63(3.35,5.95)	0.11(-0.8,1.02)
	Maldives	8.13(6.19,9.89)	1.63(1.24,1.98)	3.89(2.94,4.72)	-0.92(-8.45,7.22)
	Timor-Leste	16.44(12.9,20.95)	1.23(0.97,1.57)	3.49(2.73,4.43)	1.86(-3.06,7.03)

	Guyana	27.22(21.12,36.08)	3.53(2.74,4.68)	6.54(5.12,8.65)	0.4(-2.3,3.16)
	Namibia	40.28(30.1,51.12)	1.68(1.25,2.13)	4.1(3.08,5.16)	0.25(-2.27,2.83)
	Tuvalu	0.39(0.31,0.5)	3.29(2.59,4.2)	5.33(4.22,6.75)	-0.27(-18.38,21.85)
	Kiribati	1.65(1.3,2.11)	1.39(1.1,1.78)	4.29(3.36,5.47)	-0.23(-9.55,10.06)
	Lesotho	26.85(19.98,34.74)	1.28(0.96,1.66)	3.94(2.93,5.04)	1.82(-0.67,4.38)
	Palestine	63.87(52.25,89.15)	1.29(1.05,1.8)	4.64(3.74,6.6)	0.29(-1.72,2.33)
	Mauritania	64.41(48.45,79.57)	1.6(1.21,1.98)	4.61(3.47,5.71)	-0.31(-2.83,2.27)
	Marshall Islands	1.09(0.8,1.46)	1.91(1.41,2.58)	6.15(4.61,7.99)	0.22(-11.24,13.16)
	Mongolia	51.39(41.35,62.83)	1.52(1.22,1.85)	4.99(4.11,5.94)	-0.6(-2.56,1.4)
	Eswatini	10.74(7.77,14.04)	0.94(0.68,1.23)	3.71(2.67,4.89)	0.71(-3.38,4.98)
	Micronesia (Federated States of)	2.76(2.05,3.75)	2.7(2,3.67)	7.08(5.45,9.35)	0.6(-6.48,8.23)
	Lao People's Democratic Republic	90.36(75.33,107.88)	1.26(1.05,1.51)	3.57(2.98,4.23)	0.72(-0.96,2.42)
	Sao Tome and Principe	3.2(2.44,3.87)	1.56(1.19,1.89)	5.03(3.83,6.1)	1.13(-9.57,13.08)
	Congo	88.49(67.74,114.11)	1.68(1.29,2.17)	6.34(4.75,8.2)	-0.22(-1.74,1.32)
	Belize	10.12(7.95,13.19)	2.47(1.94,3.22)	4.53(3.56,5.89)	0.52(-5.51,6.93)
	Cabo Verde	26.66(20.71,32.78)	4.73(3.68,5.82)	6.29(4.87,7.7)	0.89(-4.97,7.11)
	Nauru	0.11(0.08,0.15)	1.04(0.77,1.42)	6.25(4.9,7.96)	0.01(-27.5,37.95)
Low-SDI	Yemen	254.81(200.89,333.85)	0.81(0.64,1.06)	3.34(2.63,4.32)	0.41(-0.6,1.43)
	Uganda	386.91(212.83,536.32)	0.94(0.52,1.3)	4.63(2.49,6.47)	0.55(-0.25,1.35)
	Afghanistan	216.48(158.95,305.66)	0.57(0.42,0.8)	3.2(2.3,4.23)	0.51(-0.41,1.44)
	Guinea	173.43(133.71,222.1)	1.37(1.06,1.76)	4.62(3.53,5.92)	0.54(-0.85,1.96)
	Bangladesh	4163.89(2642.35,5589.06)	2.61(1.66,3.51)	4.73(2.93,6.37)	1.06(0.73,1.39)
	Cambodia	231.7(183.96,290.22)	1.4(1.11,1.75)	3.16(2.47,3.94)	0.8(-0.33,1.94)
	Madagascar	322.59(205.98,429.01)	1.21(0.77,1.61)	5.67(3.62,7.54)	-0.03(-0.74,0.68)
	Mali	234.28(172.24,292.14)	1.07(0.79,1.33)	4.87(3.56,6.07)	0.39(-0.89,1.67)

	Haiti	236.94(167.5,349.44)	1.91(1.35,2.82)	5.79(4.19,8.31)	-0.25(-1.15,0.64)
	Pakistan	2712.75(2079.78,3439.71)	1.21(0.93,1.54)	4.5(3.41,5.79)	0.76(0.47,1.05)
	Ethiopia	1046.82(612.59,1377.31)	0.97(0.57,1.28)	4(2.31,5.29)	-0.72(-1.2,-0.24)
	Zimbabwe	128.75(73.45,165.2)	0.86(0.49,1.1)	3.8(1.94,4.86)	0.35(-1.08,1.79)
	Mozambique	326.23(192.1,478.38)	1.1(0.65,1.62)	5.33(3.08,7.76)	1.35(0.5,2.2)
	Angola	289.73(206.13,366.93)	0.96(0.68,1.22)	5.19(3.66,6.61)	0.66(-0.25,1.59)
	Nepal	568.78(376.89,757.31)	1.87(1.24,2.49)	4.08(2.67,5.52)	1.51(0.7,2.33)
	Burkina Faso	286.83(209.51,380.67)	1.26(0.92,1.68)	5.58(4.09,7.44)	1.87(0.72,3.03)
	Niger	145.36(97.63,195.03)	0.62(0.42,0.84)	4.07(2.7,5.48)	0.37(-1.11,1.87)
	Democratic Republic of the Congo	1156.23(677.84,1790.75)	1.32(0.77,2.04)	5.65(3.27,8.81)	0.1(-0.32,0.53)
	Malawi	185.19(111.24,242.04)	1(0.6,1.31)	4.18(2.48,5.54)	0.4(-0.72,1.54)
	Papua New Guinea	101.9(67.35,150.79)	1.03(0.68,1.53)	4.01(2.66,5.84)	0.71(-0.74,2.18)
	Benin	131.26(103.04,161.55)	1.04(0.81,1.28)	4.63(3.65,5.68)	0.42(-1.29,2.15)
	Rwanda	167.98(104.83,218.89)	1.32(0.83,1.73)	5.02(3.08,6.6)	-1(-2.08,0.09)
	Senegal	210.45(155.89,267.1)	1.39(1.03,1.76)	4.55(3.35,5.8)	0.69(-0.74,2.15)
	Cote d'Ivoire	226.45(179.15,280.65)	0.87(0.68,1.07)	4.59(3.52,5.73)	0.18(-1.05,1.43)
	United Republic of Tanzania	819.62(521.09,1064.27)	1.44(0.92,1.88)	5.11(3.21,6.7)	-0.17(-0.71,0.37)
	Somalia	110.02(53.33,162.98)	0.54(0.26,0.8)	3.7(1.69,5.5)	-0.37(-1.63,0.9)
	Chad	131.67(95.33,175.68)	0.8(0.58,1.07)	4.25(3.07,5.63)	0.55(-0.98,2.11)
	Vanuatu	4.73(3.57,6.37)	1.6(1.21,2.16)	4.32(3.2,5.74)	0.75(-6.05,8.05)
	Gambia	31.05(24.75,39.02)	1.38(1.1,1.74)	5.21(4.06,6.57)	1.05(-3.09,5.37)
	Djibouti	13.05(8.02,17.63)	1.09(0.67,1.47)	4.73(2.88,6.36)	-0.19(-4.71,4.56)
	Burundi	92.88(55.57,127.69)	0.78(0.47,1.07)	3.87(2.28,5.24)	-1.32(-2.59,-0.04)
	Bhutan	19.13(12.53,26.83)	2.54(1.66,3.56)	4.79(3.15,6.75)	1.31(-3.26,6.09)
	Eritrea	65.69(41.32,89.48)	0.98(0.62,1.33)	5.43(3.4,7.54)	0.19(-1.65,2.06)

	Comoros	15.91(11.06,20.38)	2.23(1.55,2.85)	4.43(3.04,5.71)	-0.34(-4.23,3.7)
	Guinea-Bissau	16.36(12.44,21.62)	0.86(0.65,1.14)	4.95(3.73,6.54)	0.52(-3.26,4.45)
	Liberia	50.93(38.58,65.82)	1.06(0.81,1.37)	4.36(3.32,5.64)	0.33(-2.36,3.1)
	South Sudan	86.44(50.29,125.45)	0.93(0.54,1.35)	3.75(2.18,5.39)	-0.76(-2.13,0.63)
	Central African Republic	50.21(30.35,70.32)	0.95(0.57,1.33)	4.89(2.94,6.68)	0(-1.66,1.69)
	Solomon Islands	7.95(6,11.41)	1.21(0.92,1.74)	4.59(3.47,6.03)	0.65(-4.61,6.21)
	Togo	81.34(63.29,104.53)	1.03(0.8,1.32)	4.89(3.72,6.32)	0.42(-1.61,2.49)
	Sierra Leone	83.55(64.64,110.81)	1.01(0.78,1.34)	3.98(3.07,5.29)	0.76(-1.38,2.94)

Table S3 **DALYs, all-age DALYs, age-standardized DALYs and net drift of DALYs distribution in 204 countries and territories.**

**SDI groups**	**Countries/Regions**	**Number of DALYs in 2019**	**All-age DALY rate, per 100,000 in 2019**	**Age-standardized DALY rate in 2019**	**Net drift of DALY y, % per year**
High SDI	Denmark	16963.48(13876.61,20897.4)	292.34(239.14,360.13)	137.23(111.86,170.64)	-0.19(-0.29,-0.1)
	Puerto Rico	5941.03(4677.91,7425.81)	168.71(132.84,210.87)	73.03(57.64,92.08)	-0.06(-0.24,0.12)
	Switzerland	17616.02(13769.16,22506.91)	200.75(156.91,256.48)	92.69(71.5,120.19)	-0.12(-0.22,-0.02)
	Russian Federation	312583.49(237565.76,408734.77)	213.05(161.92,278.59)	130.9(99.5,170.9)	0.4(0.36,0.43)
	Germany	333196.49(273202.68,414842.7)	392.39(321.74,488.54)	155.89(126.49,196.4)	0.48(0.32,0.64)
	Finland	16182.97(12362.9,20603.88)	292.42(223.4,372.31)	120.88(92.01,154.01)	-0.36(-0.45,-0.26)
	France	179825.4(141221.84,226711.7)	271.62(213.31,342.44)	116.24(90.34,148.61)	-0.68(-0.71,-0.64)
	Czechia	29179.05(22356.46,38059.95)	274.15(210.05,357.59)	133.84(102.33,174.44)	0.44(0.37,0.51)
	United Kingdom	199714.53(158673.56,248133.75)	297.1(236.05,369.13)	148.14(116.72,184.99)	0.07(0.03,0.12)
	San Marino	89.01(66.81,114.91)	268.92(201.83,347.17)	123.64(91.42,160.96)	-0.15(-1.53,1.25)
	Brunei Darussalam	265.1(223.71,315.07)	60.65(51.18,72.08)	127.1(110.49,146.41)	-0.15(-1.11,0.81)

Kuwait	1352.4(1020.39,1791.8)	30.55(23.05,40.48)	64.7(49.47,84.46)	0.09(-0.37,0.55)
Ireland	10588.3(8349.44,13244.83)	215.63(170.04,269.73)	137.38(107.66,171.91)	-0.67(-0.79,-0.55)
Republic of Korea	49455.1(39534.73,61931.65)	92.62(74.04,115.98)	56.82(45.46,70.8)	-0.55(-0.61,-0.48)
Taiwan (Province of China)	35192.6(26880.54,45210.09)	148.99(113.8,191.4)	87.09(66.54,111.88)	-0.15(-0.23,-0.07)
Luxembourg	1489.5(1174.86,1888.97)	240.8(189.94,305.39)	140.2(109.56,179.75)	-0.14(-0.46,0.17)
Singapore	3935.14(3015.11,5112.93)	69.43(53.2,90.22)	51.94(40.06,67.04)	-0.81(-1.03,-0.58)
Slovenia	5799.87(4421.91,7500.13)	279.61(213.18,361.58)	124.96(94.67,162.2)	-0.24(-0.39,-0.08)
United States of America	955312.32(754898.54,1198324.08)	291.27(230.17,365.37)	162.34(127.34,203.97)	1.01(0.96,1.05)
Australia	75139.22(59550.83,95423.11)	305.84(242.39,388.4)	167.01(131.41,213.42)	-0.41(-0.46,-0.37)
Iceland	799.06(638.18,1005.7)	231.7(185.05,291.61)	133.41(105.29,168.57)	-0.02(-0.46,0.41)
Canada	103817.44(78061.63,136907.83)	284.28(213.75,374.89)	142.69(106.55,188.65)	-0.26(-0.3,-0.22)
Japan	211572.24(169137.85,263444.56)	165.56(132.36,206.16)	53.15(42.54,66.74)	-1.23(-1.31,-1.15)
Slovakia	13585.64(10485.18,17529.09)	249.86(192.84,322.39)	146.25(112.91,187.67)	0.17(0.07,0.27)
New Zealand	14562.62(11633.13,18206.57)	323.93(258.76,404.98)	175.07(139.28,218.7)	-0.27(-0.38,-0.16)
Estonia	3910.98(2963.15,5111.17)	298.01(225.79,389.46)	137.68(103.3,179.54)	0.61(0.43,0.8)
Belgium	30618.93(25208.82,37942.74)	268.14(220.76,332.27)	119.42(96.46,149.63)	-0.41(-0.48,-0.34)
Qatar	752.96(565.01,974.58)	26.29(19.72,34.02)	178.86(142.32,228.96)	-0.78(-1.59,0.04)
Saudi Arabia	9816.24(7547.56,12762.88)	27.47(21.12,35.72)	85.11(66.98,105.14)	-0.03(-0.16,0.11)
Latvia	5435.79(4162.94,6988.64)	283.81(217.35,364.89)	126.88(95.98,164.14)	0.55(0.41,0.7)
Austria	31779.23(24891,40121.12)	356.42(279.17,449.98)	163.04(126.86,207.93)	0.91(0.84,0.98)
Andorra	196.67(146.13,258.98)	236.77(175.92,311.79)	133.72(99.85,176.65)	-0.39(-1.39,0.62)
Netherlands	43581.21(34852.18,55605.21)	254.02(203.14,324.1)	120.3(95.51,153.9)	-0.39(-0.46,-0.32)
United Arab Emirates	2213.92(1469.86,3300.23)	23.96(15.9,35.71)	90.02(60.43,129.24)	-0.17(-0.92,0.58)
Lithuania	8223.74(6208.5,10642.57)	294.31(222.19,380.88)	133.1(99.48,174.78)	0.4(0.28,0.52)
Bermuda	108.21(87.59,134.36)	169(136.8,209.84)	76.91(62.35,95.72)	-0.98(-2.25,0.31)

	Cyprus	2775.37(2280.37,3420.1)	211.3(173.61,260.39)	150.07(124.21,182.95)	-1.02(-1.29,-0.75)
	Guam	195.63(153.57,251.02)	114.65(90,147.12)	105.84(83.07,136.07)	-0.88(-1.88,0.14)
	Norway	14446.2(11280.6,17668.42)	270.08(210.9,330.32)	138.06(108.02,170.33)	-0.4(-0.5,-0.3)
	Sweden	44218.22(29996.49,56073.2)	432.56(293.43,548.52)	188.07(132.83,241.02)	0.57(0.48,0.67)
	Monaco	130(102.12,162.46)	345.99(271.81,432.39)	122.34(95.13,155.06)	-0.29(-1.33,0.75)
High-middle SDI	Dominica	109.36(91.76,130.75)	159.23(133.6,190.38)	119.33(100.15,142.62)	0.5(-0.68,1.68)
	Croatia	9496.23(7230.7,12315.71)	223.55(170.22,289.92)	103.77(78.57,135.05)	-0.31(-0.43,-0.19)
	Ukraine	121590.95(94110.16,156579.54)	276.08(213.68,355.52)	155.92(121.12,200.19)	0.4(0.35,0.45)
	Hungary	26679.11(20367.15,34890.48)	275.77(210.53,360.65)	131.8(100.04,172.95)	-0.36(-0.42,-0.29)
	Argentina	53856.48(44271.99,66837.04)	119.38(98.13,148.15)	96.73(79.44,120.36)	0.14(0.07,0.21)
	Saint Kitts and Nevis	74.93(63.15,93.92)	125.92(106.12,157.83)	146.73(125.35,182.39)	-0.22(-1.62,1.21)
	North Macedonia	4437.93(3516.72,5600.81)	206.15(163.36,260.17)	159.84(129.27,197.35)	-0.03(-0.21,0.15)
	Kazakhstan	21271.55(15654.83,29563.72)	115.66(85.12,160.74)	146.76(110.39,207.2)	0.43(0.33,0.54)
	Romania	45679.99(34324.7,59763.3)	237.46(178.43,310.67)	116.9(87.37,153.71)	-0.56(-0.62,-0.5)
	Chile	27026.11(22203.26,33915.36)	148.51(122.01,186.36)	112.55(92.57,141.1)	0.15(0.07,0.24)
	Poland	106704.49(83133.33,135428.61)	277.63(216.3,352.36)	146.05(113.54,186.37)	0.38(0.32,0.44)
	Montenegro	2377.22(1955.06,2958.97)	383.21(315.16,476.99)	250.62(205.82,313.72)	0.56(0.32,0.81)
	Greece	32484.94(25370.94,44510.36)	314.25(245.43,430.59)	119.96(92.52,160.23)	-0.53(-0.59,-0.46)
	Malaysia	25181.15(19096.8,32307.23)	80.45(61.01,103.21)	109.7(84.5,139.98)	0.42(0.33,0.52)
	United States Virgin Islands	195.51(166.05,230.75)	188.01(159.69,221.91)	115.41(98.6,135.98)	0.9(-0.08,1.89)
	Lebanon	4427.76(3335.65,5559.39)	85.53(64.43,107.38)	86.47(65.25,108.85)	-0.08(-0.28,0.13)
	Serbia	21639.85(16702.31,28023.76)	247.4(190.95,320.39)	139.63(108.59,179.45)	-0.1(-0.21,0)
	Antigua and Barbuda	99.77(84.49,121.41)	112.75(95.48,137.21)	114.01(96.92,139.06)	0.35(-0.93,1.65)
	Israel	14139.65(11108.66,18197.46)	151.88(119.32,195.47)	116.32(90.74,150.52)	-0.83(-0.94,-0.72)

	Bulgaria	23544.65(18077.53,31008.02)	339.52(260.69,447.15)	156.83(120.82,204.6)	0.21(0.12,0.3)
	Italy	189786.72(152217.44,238078.33)	314.67(252.38,394.74)	115.84(91.37,147.56)	-0.81(-0.84,-0.78)
	Barbados	456.48(375.77,561.4)	153.3(126.19,188.53)	92.33(76.17,113.36)	0.15(-0.47,0.78)
	Trinidad and Tobago	1461.19(1115.48,1832.71)	105.31(80.4,132.09)	82.78(63.32,104)	0.26(-0.1,0.61)
	Bahamas	355.1(292.67,441.47)	94.2(77.64,117.12)	104.43(86.3,129.47)	0.21(-0.51,0.94)
	Bosnia and Herzegovina	8538.36(6614.32,10978.2)	258.74(200.44,332.67)	145.72(113.24,186.08)	0.28(0.15,0.41)
	Malta	1130.62(887.26,1443.69)	257.41(202.01,328.69)	114.9(89.45,147.87)	-0.29(-0.67,0.1)
	Jordan	4032.74(3168.88,5062.98)	34.66(27.23,43.51)	85.19(68.51,104.14)	-0.29(-0.56,-0.03)
	Belarus	22656.34(16907.57,29587.02)	238.47(177.96,311.42)	138.54(103.48,181.55)	0.63(0.56,0.71)
	Seychelles	106.17(79.7,136.82)	103.94(78.03,133.95)	108.85(82.2,139.96)	0.03(-1.12,1.19)
	Portugal	29239.19(22636.03,38168.08)	274.51(212.52,358.34)	110.87(84.5,145.72)	-0.82(-0.89,-0.75)
	Cook Islands	30.02(23.84,37.74)	166.89(132.53,209.84)	124.97(99.65,157.03)	-0.1(-2.39,2.23)
	Libya	3094.51(2247.74,3981.27)	45.94(33.37,59.11)	70.76(51.83,91.33)	0.26(0.01,0.51)
	American Samoa	55.63(44.34,69.09)	100.22(79.89,124.47)	131.02(104.81,162.47)	0.09(-1.72,1.93)
	Turkey	61728.56(47405.24,79020.62)	75.87(58.27,97.13)	74.82(57.84,95.3)	-0.81(-0.96,-0.67)
	Bahrain	945.88(716.72,1174.38)	65.56(49.68,81.4)	165.78(129.43,198.77)	2.32(1.46,3.19)
	Spain	135053.54(108181.2,168600.74)	293.46(235.07,366.35)	123.88(97.64,157.71)	-0.6(-0.67,-0.54)
	Oman	1093.97(871.07,1367.49)	23.87(19,29.83)	120.67(95.12,145.85)	0.06(-0.33,0.45)
	Greenland	127.54(102.92,157.1)	226.99(183.16,279.59)	214.86(175.09,264.43)	-0.17(-1.31,0.98)
	Niue	2.78(2.2,3.51)	166.44(131.57,209.86)	130.4(103.33,164.13)	0.04(-5.77,6.21)
	Northern Mariana Islands	63.67(51.41,77.66)	149.82(120.97,182.74)	153.03(125.88,182.92)	0.4(-1.79,2.63)
	Palau	18.44(14.24,23.78)	102.41(79.06,132.03)	104.56(81.28,132.36)	-0.03(-3.2,3.24)
Middle SDI	Costa Rica	3732.7(2929.94,4709.27)	79.14(62.12,99.84)	73.08(57.4,92.39)	0.06(-0.18,0.3)
	Iran (Islamic Republic of)	47997.33(37710.42,60548.24)	56.94(44.73,71.83)	75.3(59.93,94.58)	-0.08(-0.15,-0.02)
	Gabon	998.85(743.12,1271.32)	57.08(42.46,72.65)	120.62(90.4,152.56)	0.09(-0.29,0.48)

Armenia	4990.09(3809.22,6603.95)	165.25(126.15,218.7)	122(93.57,160.53)	0.47(0.3,0.64)
Azerbaijan	9148.82(6882.86,12076.62)	89.01(66.96,117.49)	131.39(103.7,165.3)	0.89(0.76,1.02)
Grenada	93.92(79.51,116.28)	91(77.03,112.65)	98.21(83.59,121.68)	0.05(-1.12,1.24)
Iraq	18169.49(14107.4,24797.11)	43.14(33.49,58.87)	96.62(75.38,137.25)	0.42(0.31,0.53)
Tokelau	1.58(1.25,1.98)	112.09(88.32,140.23)	127.86(101.81,159.67)	0.14(-7.74,8.71)
Sri Lanka	21409.12(16192.36,28046.27)	97.96(74.09,128.33)	94.03(71.76,122.08)	0.37(0.28,0.47)
China	1729840.35(1309685.75,2226444.82)	121.62(92.08,156.53)	97.08(75.21,123.12)	-0.03(-0.06,0)
Georgia	9930.08(7924.59,12305.77)	270.96(216.24,335.79)	155.57(122.96,193.37)	1(0.88,1.11)
Botswana	817.08(625.35,1035.9)	34.94(26.74,44.29)	79.07(60.67,100.19)	0.07(-0.4,0.54)
Thailand	94883.83(71373.89,123672.57)	135.33(101.8,176.39)	95.56(72.27,124.94)	-0.19(-0.24,-0.15)
Uruguay	5732.92(4514.54,7038.49)	166.84(131.38,204.84)	93.87(73.79,116.24)	0.01(-0.16,0.17)
South Africa	30444.83(24786.07,37352.08)	54.77(44.59,67.19)	80.28(66.26,97.2)	0.31(0.24,0.38)
Saint Lucia	221.86(184.65,270.02)	127.05(105.74,154.63)	110.01(91.89,133.88)	-0.62(-1.54,0.32)
Mexico	99070.24(81768.34,122853.58)	79.29(65.45,98.33)	91.86(75.82,114.22)	0.02(-0.05,0.1)
Fiji	814.89(643.4,1020.65)	89.43(70.61,112.01)	133.13(107.11,164.82)	0.42(-0.09,0.94)
Jamaica	2990.61(2385.89,3852.78)	106.4(84.88,137.07)	93.1(74.03,120.71)	0.47(0.23,0.71)
Indonesia	192969.27(143384.63,249119.61)	74.37(55.26,96.01)	113.21(86.59,143.72)	0.49(0.46,0.52)
Egypt	40892.81(29898.61,53997.19)	41.28(30.18,54.5)	85.85(62.32,110.67)	0.37(0.31,0.44)
Paraguay	4958.83(3935.88,6206.71)	71.55(56.79,89.56)	95.45(75.89,119.73)	0.38(0.19,0.58)
Saint Vincent and the Grenadines	132.49(110.91,163.77)	117.1(98.03,144.74)	108.12(91.09,133.85)	0.16(-0.96,1.29)
Algeria	24352.38(18955.53,30289.51)	58.19(45.3,72.38)	91.18(71.78,111.62)	-0.01(-0.21,0.19)
Suriname	494.77(407.86,596.59)	85.91(70.82,103.59)	89.87(73.93,107.84)	0.17(-0.43,0.77)
Panama	3109.2(2442.8,3933.46)	74.73(58.71,94.54)	73.5(57.6,93.12)	0.18(-0.09,0.44)
Turkmenistan	4314.58(3288.56,5581.36)	84.88(64.7,109.8)	130.65(100.58,167.74)	0.58(0.38,0.79)
Peru	17548.16(13727.83,21938.85)	51.62(40.38,64.53)	54.39(42.48,68.2)	-0.52(-0.62,-0.41)

	Mauritius	1703.4(1311.74,2192.05)	133.43(102.75,171.7)	103.71(80.14,133)	-0.05(-0.37,0.28)
	Albania	5202.1(3927.81,6861.05)	191.23(144.39,252.21)	120.14(91.29,157.68)	0.11(-0.07,0.3)
	Uzbekistan	20563.11(15838.54,26570.48)	61.06(47.03,78.9)	164.01(134.73,200.8)	2.16(2.05,2.27)
	Brazil	230116.29(189167.01,279885.9)	106.21(87.31,129.18)	102.5(84.33,124.48)	0.59(0.52,0.65)
	Tonga	89.86(70.47,114.79)	87.8(68.85,112.15)	117.17(91.79,150.11)	0.27(-0.96,1.51)
	Ecuador	9551.22(7759.15,11645.32)	54.3(44.12,66.21)	72.69(59.44,88.27)	1.13(0.97,1.3)
	Samoa	169.92(133.49,212.91)	80.4(63.16,100.74)	129.58(101.89,162.28)	-0.05(-0.96,0.87)
	Cuba	15260.84(12275.62,18912.95)	134.36(108.07,166.51)	75.58(60.87,94.11)	0.07(-0.04,0.18)
	Equatorial Guinea	428.92(304.47,580.94)	30.21(21.44,40.92)	119.46(85.19,163.85)	1.08(0.31,1.87)
	Colombia	38546.42(30513.54,48094.65)	80.68(63.87,100.67)	70.54(55.84,88.37)	-0.06(-0.13,0.02)
	Tunisia	9334.85(7126.81,11851.38)	80.67(61.59,102.42)	82.77(63.74,104.79)	0.28(0.13,0.42)
	Republic of Moldova	7004.6(5188.67,9336.29)	189.92(140.68,253.14)	120.03(89.33,159.45)	-0.17(-0.31,-0.04)
Low-middle SDI	Maldives	250.32(188.11,326.7)	50.22(37.74,65.55)	99.21(75.87,127.04)	-0.18(-1.21,0.87)
	Philippines	62918.87(47210.48,82567.9)	56.11(42.1,73.63)	97.53(74.38,126.25)	0.58(0.53,0.64)
	Ghana	9079.76(7102.44,11399.43)	28.79(22.52,36.15)	76.96(61.54,94.77)	0.33(0.18,0.48)
	Myanmar	39276.47(29923.35,50422.44)	71.83(54.73,92.22)	99.7(76.99,126.11)	0.27(0.21,0.34)
	Sudan	11805.94(8970.14,15214.9)	28.93(21.98,37.28)	77.82(59.82,99.82)	0.26(0.14,0.38)
	Zambia	4872.83(3512.51,6010.66)	26.72(19.26,32.96)	99.95(72.15,122.67)	1.23(1.01,1.44)
	Democratic People's Republic of Korea	28154.85(21698.8,35963.55)	107.33(82.72,137.09)	96.57(75.12,122.93)	-0.02(-0.09,0.06)
	Dominican Republic	7735.88(6211.46,9457.61)	71.09(57.08,86.91)	90.95(73.12,111.08)	0.94(0.77,1.11)
	Guatemala	6486.62(5133.1,8087.88)	36.49(28.88,45.5)	67.39(53.14,83.25)	-0.5(-0.68,-0.31)
	El Salvador	4684.92(3712.21,5707.77)	74.89(59.34,91.23)	73.52(58.16,90.41)	0.15(-0.05,0.35)
	Honduras	4522.73(3678.7,5645.35)	46.08(37.48,57.52)	92.63(74.86,116.14)	1.17(0.95,1.39)
	Timor-Leste	653.87(484.72,868.68)	48.99(36.31,65.08)	96.73(72.81,126.94)	0.69(0.07,1.32)
	Guyana	557.84(445.99,701.03)	72.38(57.87,90.96)	108.96(88.07,136.65)	0.26(-0.25,0.78)

	Namibia	958.63(746.27,1199.6)	39.89(31.05,49.92)	80.95(63.56,100.37)	0.25(-0.17,0.68)
	Kyrgyzstan	4255.79(3177,5591.1)	65.12(48.61,85.55)	108.95(82.81,140.7)	0.43(0.26,0.6)
	Tajikistan	4836.79(3695.93,6222.41)	50.95(38.94,65.55)	147.07(117.62,182.69)	0.85(0.68,1.01)
	India	1024772.24(786262.3,1309013.29)	73.69(56.54,94.13)	105.66(81.67,132.84)	0.16(0.07,0.26)
	Tuvalu	12.19(9.56,15.35)	103.28(81.02,130.14)	135.04(106.88,169.56)	-0.09(-3.66,3.61)
	Nicaragua	3507.81(2939.63,4158.3)	53.88(45.15,63.87)	99.89(84.02,117.79)	0.7(0.45,0.94)
	Viet Nam	91403.58(69114.94,116524.56)	94.84(71.72,120.91)	114.08(86.71,144.54)	0.53(0.48,0.57)
	Kenya	12426.17(9173.09,15663.71)	24.74(18.26,31.19)	78.47(56.47,99.88)	1.25(1.11,1.38)
	Kiribati	69.73(54.27,87.98)	58.78(45.75,74.17)	124.71(98.8,154.5)	-0.07(-2.04,1.94)
	Nigeria	55780.5(43338.08,69219.95)	25.97(20.17,32.22)	87.69(68.73,107.82)	-0.2(-0.25,-0.15)
	Lesotho	774.12(590.52,976.5)	37.01(28.23,46.69)	79.68(61.39,99.6)	1.02(0.62,1.42)
	Palestine	1684.59(1342.55,2146.63)	33.99(27.09,43.31)	92.92(74.6,117.47)	0.2(-0.13,0.53)
	Morocco	22499.69(17709.74,28188.8)	62.58(49.26,78.41)	89.32(70.27,110.18)	0.42(0.33,0.51)
	Mauritania	1410.26(1110.28,1756.75)	35.13(27.66,43.76)	83.45(66.01,103.11)	-0.06(-0.4,0.28)
	Marshall Islands	39.64(30.14,51.39)	69.74(53.02,90.41)	147.09(114.96,187.12)	0.18(-2.21,2.63)
	Venezuela (Bolivarian Republic of)	21986.1(17333.4,27579.23)	78.33(61.75,98.26)	81.03(64.06,101.17)	0.04(-0.06,0.14)
	Mongolia	2208.27(1648.98,2885.91)	65.19(48.68,85.19)	127.39(98.98,161.65)	-0.04(-0.31,0.24)
	Eswatini	331.78(256.78,425.25)	29.05(22.48,37.23)	76.11(59.09,96.47)	0.37(-0.32,1.07)
	Micronesia (Federated States of)	86.17(64.76,110.25)	84.39(63.42,107.96)	156.79(122.38,198.07)	0.36(-1.06,1.81)
	Lao People's Democratic Republic	3503.88(2643.27,4555.07)	48.95(36.93,63.63)	100.43(77.33,127.89)	0.31(0.08,0.55)
	Sao Tome and Principe	72.3(57.53,90.15)	35.2(28.01,43.89)	90.16(72.25,111.18)	0.62(-0.81,2.06)
	Bolivia (Plurinational State of)	6739.19(5107.17,8988.03)	56.1(42.52,74.83)	92.97(70.89,121.81)	0.41(0.23,0.59)
	Cameroon	8127.53(6452.36,9994.21)	27.93(22.17,34.34)	97.63(78.11,119.12)	0.43(0.28,0.59)
	Congo	2163.88(1681.85,2658.36)	41.09(31.94,50.48)	113.56(90.29,139.8)	-0.11(-0.43,0.21)
	Syrian Arab Republic	8477.17(6460.34,10815.65)	58.5(44.58,74.64)	90.57(69.49,113.06)	0.08(-0.06,0.22)

	Belize	201.26(167.15,246.5)	49.08(40.76,60.11)	83.32(69.27,101.13)	0.42(-0.6,1.46)
	Cabo Verde	414.1(335.75,495.64)	73.48(59.58,87.95)	100.8(81.8,121.1)	0.55(-0.17,1.27)
	Nauru	4.58(3.46,6.03)	43.43(32.77,57.13)	150.32(117.66,190.06)	0.03(-6.17,6.63)
Low-SDI	Vanuatu	186.15(140.69,245.4)	63.2(47.76,83.31)	125.63(95.86,161.32)	0.34(-0.88,1.57)
	Yemen	7951.08(5991.9,10322.47)	25.24(19.02,32.77)	77.67(59.98,99.56)	0.25(0.09,0.41)
	Uganda	7936.56(4967.39,10423.24)	19.3(12.08,25.35)	74.91(46.54,98.31)	0.39(0.24,0.54)
	Gambia	674.91(540.23,833.19)	30.05(24.05,37.1)	89.47(71.89,109.22)	0.49(-0.08,1.08)
	Afghanistan	7078.63(5363.65,9385.66)	18.49(14.01,24.52)	76.41(59.48,98.79)	0.33(0.19,0.48)
	Djibouti	312.82(219.1,414.5)	26.01(18.22,34.46)	77.52(53.58,100.61)	-0.03(-1.02,0.97)
	Guinea	3687.27(2869.87,4581.26)	29.16(22.7,36.24)	82.98(65.41,102.29)	0.35(0.16,0.55)
	Burundi	2221.81(1553.42,2911.06)	18.62(13.02,24.39)	67.89(46.25,88.39)	-0.92(-1.16,-0.67)
	Bangladesh	118576.27(88485.66,151181.47)	74.45(55.56,94.93)	105.11(78.77,133.15)	0.47(0.37,0.57)
	Bhutan	539.15(403.08,691.18)	71.48(53.44,91.64)	111.59(83.06,143.06)	0.61(-0.03,1.25)
	Eritrea	1504.78(1061.44,1946.59)	22.42(15.82,29.01)	85.11(59.17,111.05)	0.19(-0.23,0.62)
	Cambodia	9447.24(7051.6,12396.15)	56.9(42.47,74.66)	95.56(72.43,124.5)	0.49(0.34,0.65)
	Comoros	302.29(225.51,372.26)	42.32(31.57,52.11)	72.7(54.24,89.34)	-0.25(-0.98,0.5)
	Guinea-Bissau	425.39(329.25,532.84)	22.38(17.32,28.03)	87.98(69.13,109.87)	0.32(-0.27,0.92)
	Liberia	1210.34(941.48,1528.36)	25.27(19.66,31.91)	80.71(63.04,101.6)	0.15(-0.2,0.5)
	Madagascar	7346.08(5263.8,9513.1)	27.52(19.72,35.64)	93.69(65.65,120.29)	-0.01(-0.16,0.13)
	Mali	5352.52(4172.71,6727.15)	24.42(19.04,30.69)	84.82(66.2,105.27)	0.25(0.08,0.43)
	Haiti	5242.69(3903.25,7241.81)	42.27(31.47,58.39)	98.37(74.55,135.09)	-0.18(-0.35,0)
	Pakistan	95095.11(72529.94,121182.68)	42.44(32.37,54.08)	112.61(87.07,141.19)	0.34(0.3,0.38)
	Ethiopia	21970.43(15023.1,27862.92)	20.42(13.96,25.9)	67.64(45.72,85.46)	-0.4(-0.5,-0.3)
	Zimbabwe	3554.6(2689.93,4477.65)	23.68(17.92,29.83)	71.95(54.32,89.59)	0.17(-0.03,0.36)
	Mozambique	6734.1(4412.48,9337.96)	22.81(14.94,31.62)	84.39(54.17,116.32)	1.01(0.84,1.17)

	Angola	7877.71(5868.36,9852.64)	26.14(19.47,32.69)	99.37(74.9,123.73)	0.5(0.31,0.68)
	South Sudan	1882.63(1284.54,2528.33)	20.28(13.84,27.24)	64.93(43.6,87.68)	-0.5(-0.76,-0.24)
	Nepal	18597.41(14007.08,24134.1)	61.14(46.05,79.35)	100.29(75.42,129.72)	0.62(0.51,0.73)
	Burkina Faso	6469.56(4940.52,8115.18)	28.51(21.77,35.76)	97.29(75.09,121.7)	0.97(0.79,1.14)
	Niger	4124.81(3042.49,5354.71)	17.71(13.06,22.99)	77.14(56.98,98.57)	0.2(-0.03,0.42)
	Democratic Republic of the Congo	27823.62(18995.34,38914.79)	31.74(21.67,44.39)	104.42(70.43,145.61)	0.03(-0.06,0.12)
	Malawi	3897.12(2642.11,4942.57)	21.13(14.33,26.8)	70.07(46.97,87.9)	0.32(0.11,0.53)
	Papua New Guinea	4112(3014.2,5473.37)	41.68(30.55,55.47)	111.18(83.7,145.8)	0.39(0.13,0.65)
	Central African Republic	1464.84(1042.97,1934.3)	27.64(19.68,36.5)	97.78(69.1,127.48)	-0.01(-0.37,0.35)
	Benin	3063.42(2441.86,3764.37)	24.19(19.28,29.72)	83.71(67.66,102.29)	0.31(0.07,0.54)
	Rwanda	3485.7(2447.7,4372.87)	27.47(19.29,34.46)	79.35(54.68,99.08)	-0.76(-0.98,-0.55)
	Senegal	4824.62(3767.07,6078.66)	31.88(24.89,40.17)	82.46(64.54,103.12)	0.3(0.11,0.49)
	Solomon Islands	315.25(236.75,425.21)	48.08(36.11,64.85)	127.94(98.35,163.44)	0.35(-0.79,1.51)
	Togo	2125.48(1640.19,2656.65)	26.83(20.71,33.54)	86.61(68.31,106.93)	0.2(-0.11,0.5)
	Coe d'Ivoire	5978.89(4698.03,7555.11)	22.84(17.95,28.87)	83.68(66.98,103.23)	0.14(-0.05,0.32)
	Sierra Leone	2194.46(1671.04,2770.92)	26.49(20.17,33.45)	79.39(61.49,100.19)	0.26(0,0.52)
	United Republic of Tanzania	16456.79(11438.67,20868.64)	29.01(20.16,36.78)	84.74(58.4,106.72)	-0.1(-0.2,0.01)
	Somalia	2877.89(1776.8,3966.21)	14.15(8.73,19.5)	64.46(37.68,89.04)	-0.24(-0.51,0.03)
	Chad	3320.1(2538.37,4229.84)	20.25(15.48,25.79)	79.93(61.33,100.2)	0.36(0.14,0.57)
